# Network pharmacology and UPLC/MS/MS metabolic profiling unveil the anti-inflammatory potential of *Trifolium alexandrinum*

**DOI:** 10.1038/s41538-025-00459-y

**Published:** 2025-06-14

**Authors:** Rahma SR. Mahrous, Hoda Fathy, Doaa A. Ghareeb, Ali S. Abdel‑Hamid, Reham S. Ibrahim

**Affiliations:** 1 Department of Pharmacognosy, Faculty of Pharmacy, Alexandria university, Alexandria, Egypt; 2 Bio-Screening and Preclinical Trials Lab, Biochemistry Department, Faculty of Science, Alexandria University, Alexandria, Egypt; 3 Center of Excellence for Drug Preclinical Studies (CE-DPS), Pharmaceutical and Fermentation Industry Development Center, City of Scientific Research & Technological Applications, New Borg El Arab, Alexandria, Egypt; 4 Research Projects unit, Pharos University in Alexandria; Canal El Mahmoudia Street, Beside Green Plaza Complex, 21648 Alexandria, Egypt

**Keywords:** Drug discovery, Biochemical networks

## Abstract

*Trifolium alexandrinum*, commonly known as berseem clover, has long been used in traditional medicine for its diverse therapeutic properties. In this study, we explore the anti-inflammatory potential of *T. alexandrinum* through an integrated approach combining network pharmacology and LC-MS/MS metabolic profiling. The ethanolic extract of *T. alexandrinum* was fractionated and analyzed, revealing a rich profile of phytoconstituents, including flavonoids, isoflavonoids, triterpenoid glycosides, and purine nucleosides. Network pharmacology analysis identified key bioactive compounds, such as tryptophan and adenosine, which exhibited strong interactions with inflammation-related genes, including TNF-α, IL-6, IL-1β, and INF-γ, as demonstrated from the “compound-target-pathway” constructed network. The arachidonic acid metabolism pathway, which plays a pivotal role in inflammation, was the top-listed pathway in the network. For the sake of confirmation, tryptophan and adenosine were isolated from the butanol fraction, and their structures were elucidated using ^1^H-NMR, ^13^C-DEPTQ, and HRESI-MS. In vitro studies using LPS-stimulated WI38 human fibroblast cells demonstrated that the butanol fraction of the extract significantly reduced the expression of pro-inflammatory cytokines, with adenosine and tryptophan showing particularly potent anti-inflammatory effects comparable to the synthetic drug piroxicam. These findings suggest that *T. alexandrinum* and its constituents, particularly polar compounds in the butanol fraction, hold promise as natural anti-inflammatory agents. This study not only elucidates the molecular mechanisms underlying the anti-inflammatory properties of *T. alexandrinum* but also highlights its potential as a functional food ingredient with both nutritional and therapeutic benefits.

## Introduction

*Trifolium alexandrinum*, family Fabaceae, known in Arabic as Berseem or Egyptian clover, is an annual plant cultivated in Egypt and is considered one of the most spreading fodder species for cattle^1^. The plant seeds have been used by the Egyptians as antidiabetic since ancient times^2^. Berseem clover is cultivated in many countries, for instance, Syria, Turkey, Iran, Pakistan, South America, India, and others^3^. Ethnopharmacological evaluation has been performed for different *Trifolium* species. Yet, *Trifolium pratense* (red clover) is the most studied species among others. Red clover is currently available and marketed as a dietary supplement for its estrogenic effects to be used in menopause and osteoporosis^4^.

*T. alexandrinum* is used in folk medicine for divergent illnesses. In Pakistan, the entire plant is used for wound healing. Dried flowers are used for diseases of the respiratory system, such as asthma. In Kurdistan (Iraq), leaves decoction is used for colic. Egyptian clover is rich in phytoconstituents. Aerial parts contain flavonoids; the isoflavone group, which exists in their free and glycosidic forms^4,5^. Chalcones, triterpene saponins, and megastigmane glycosides were identified in *T. alexandrinum* seeds^6^. Moreover, the nucleoside xanthosine was isolated from the plant seeds^7^.

Recent in vitro and in vivo studies showed a wide range of biological activities of *T. alexandrinum*, where the extract of aerial parts had strong antioxidant and antimicrobial actions. Hepatoprotective effects were reported for the root and flower extracts. The plant extract showed estrogenic and hepatoprotective effects^1,5^. Additionally, the plant extract and its flavonoids hesperetin and quercetin exhibited antidiabetic action with improvement in insulin levels, serum glucose, and lipid metabolism. The same study identified anti-inflammatory activity as a potential mechanism underlying the antidiabetic effects of *T. alexandrinum*, with the plant extract significantly suppressing tumor necrosis factor-alpha (TNF-α) and interleukin-6 (IL-6) expression in the pancreatic tissue of diabetic rats^8^. Yet, the anti-inflammatory potential of berseem and its phytoconstituents hasn’t been thoroughly investigated.

Inflammation, including acute and chronic ones can be considered a part of the body’s defense mechanism responding to biological stimuli, injury or autoimmune response. It is considered a healing mechanism. Although inflammation is an essential complex process for human health, excessive uncontrolled inflammation may cause many diseases^7^. Rheumatoid arthritis, type 2 diabetes, Alzheimer’s, atherosclerosis, cardiovascular diseases, and cancers are a few examples of diseases related to chronic inflammatory responses^9^.

Inflammatory cascade in the body is triggered by different mediators that are produced through either internal or external induction^10^. Lipopolysaccharide (LPS), the major component of the outer cell membrane of gram-negative bacteria, is a major inducer of inflammation through binding to Toll-like receptor 4 (TLR4) that is located on several cell types of the immune system, mainly macrophages and monocytes. This, in turn, produces mediators through activation of complex signaling pathways, which invoke an inflammatory response^9^. Tumor necrosis factor-alpha (TNF-α), interleukin-1 beta (IL-1β), interleukin-6 (IL-6), interleukin-8 (IL-8), and others are examples of the inflammatory mediators (cytokines) produced following stimulating TLR4 signaling pathways. Type I interferons are also produced in response to activation of these pathways^9,11^. Increased oxidative stress and cell damage is noticed due to the induction of nitric oxide NO and reactive oxygen species ROS by LPS. Unregulated inflammatory response leads to exaggerated production of the inflammatory mediators, leading to overstimulation of the immune response. This results in chronic inflammation, which ultimately results in the progression of metabolic dysfunctions and many chronic diseases^12^. Treatment of inflammation is a complicated process which involves controlling many signaling pathways. Downregulation of inflammatory cytokines is an interesting approach in treatment as they represent the principle inflammatory mediators involved in the acute and chronic inflammatory process^13^.

The utilization of natural products as multitargeted anti-inflammatory agents has evolved over the past few years. The complex matrix of these natural products as plant extracts makes them perfect candidates in the treatment of complex diseases such as inflammation owing to their synergistic potential of their multicomponent^14^.

Network pharmacology provides an effective means towards identifying the mechanism of drug action, especially for complex diseases. This multidisciplinary field makes use of the advancements in systems biology, polypharmacology, and bioinformatics^11^. This field is vastly spreading in the drug discovery process and considers a network mode of “multiple targets, multiple effects, complex diseases” as its main core. Network pharmacology offers a unique framework to explore the molecular intricacies of herbal extracts and their relationships with different targets and complex disorders in a systematic manner^15^.

Metabolomics is a powerful approach for analyzing the bioactive scaffold of herbal extracts. The integration of this technique with network pharmacology enables a thorough investigation of the molecular mechanisms that underpin the therapeutic effects of these extracts in a holistic manner^15^.

In this study, the anti-inflammatory potential of *T. alexandinum* was investigated through the utilization of metabolic profiling and network pharmacology analysis. Metabolic profiling was performed to identify potential phytoconstituents. This was integrated into a network pharmacology analysis to clarify the molecular mechanisms by which the plant constituents contribute to the treatment of the complex nature of inflammation. Then, active biomarkers were isolated from *T. alexandinum* extract. Finally, the anti-inflammatory mechanism of the plant extract and isolated compounds were evaluated in vitro for their effects in reducing inflammatory cytokines (TNF-α, IL-1β, and IL-6) and interferons INF-γ gene expression levels in LPS-stimulated WI38 human fibroblast cells.

## Results and discussion

### LC-MS/MS analysis of *T. alexandrinum* ethanolic extract and solvent fractions

**Fig. 1:**
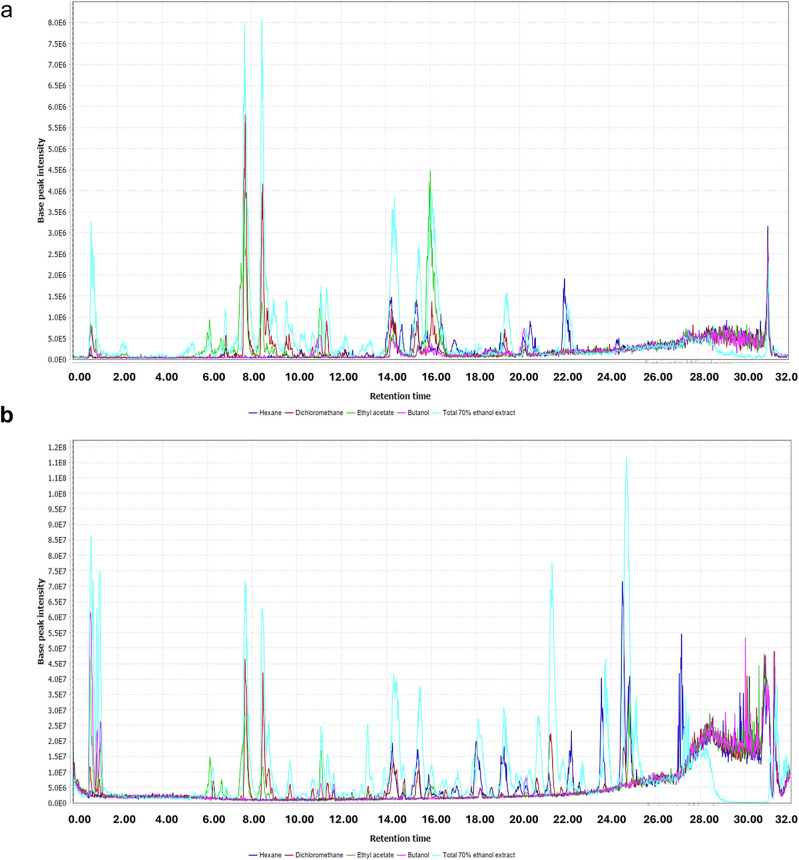
UPLC-MS base peak chromatogram of *T. alexandrinum* extract and solvent fractions. Negative ion mode (**a**) and positive ion mode (**b**).

**Fig. 2:**
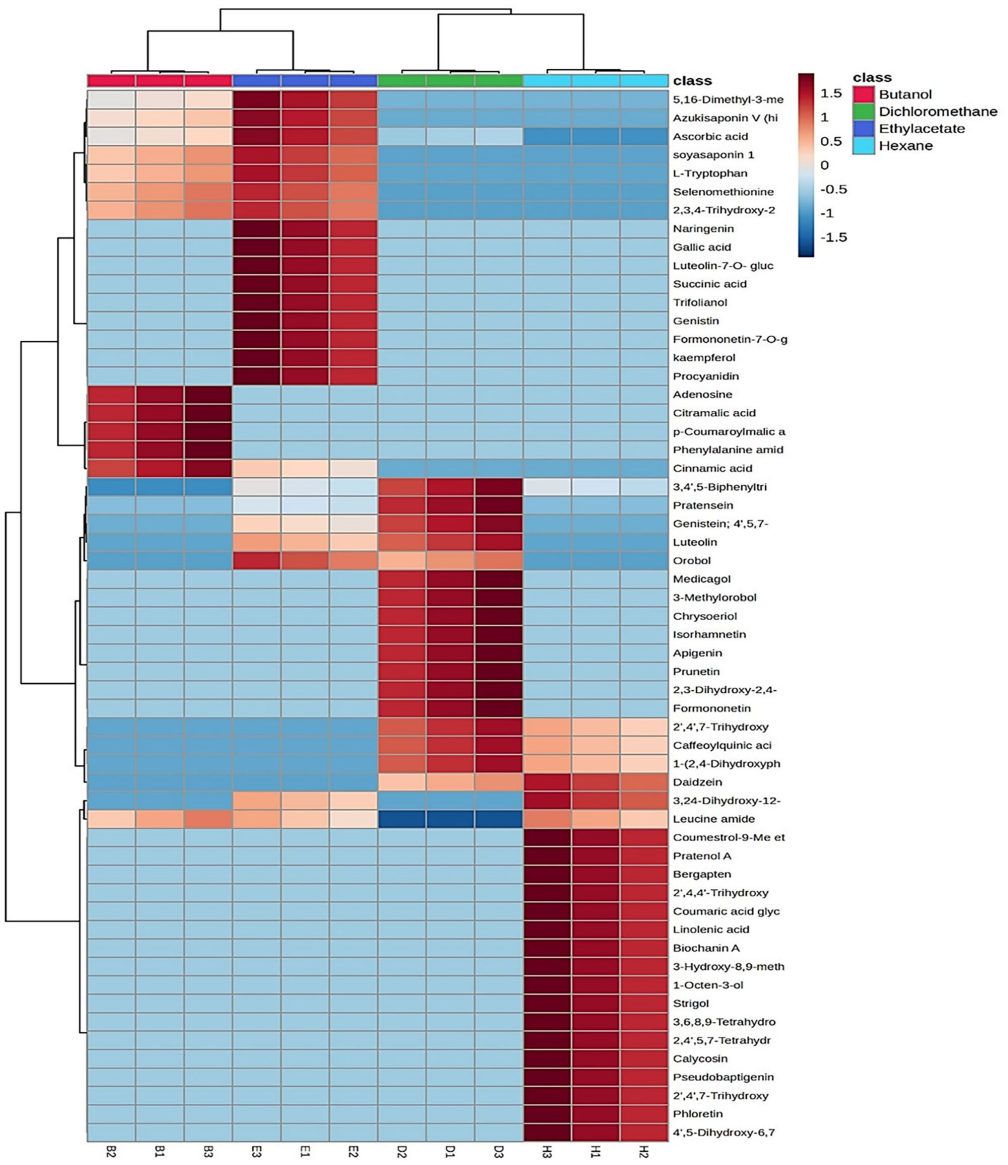
HCA dendrogram-heat map of relative distribution of identified compounds in the four solvent fractions of *T. alexandrinum*.

Metabolic profiling of *T. alexandrinum* ethanolic extract and solvent fractions using UPLC-MS/MS revealed its richness in phytoconstituents with flavonoids of different classes predominating (Fig. 1). A total of 57 compounds were identified (Table 1), including flavonoid and isoflavonoid glycosides such as luteolin O-glucoside, genistin, and kaempferol 3-*O*-galactoside. Isoflavones such as daidzein, pratensein, genistein, formononetin, pseudobaptigenin, prunetin, and orobol (and its methyl ether) were also identified. Flavones, including luteolin and apigenin, along with chalcones and coumestans like coumestrol 9-Me ether, were detected. Furthermore, triterpenoid glycosides were identified, including azukisaponin V, soyasaponin I, and 3,24-dihydroxy-12-oleanen-29-oic acid 3-*O*-pentosylhexoside. Additionally, aliphatic and aromatic acids, amino acid derivatives, and purine nucleosides such as adenosine were also present in the extract and fractions (Fig. S1). The distribution of annotated phytoconstituents in the four solvent fraction is depicted in heat map in (Fig. 2), where the change of color from light blue to brick red indicates increase in relative quantity. Hierarchical clustering analysis (HCA) revealed that butanol and ethyl acetate fractions were clustered together, indicating proximity in their composition, especially in the abundance of polar compounds. Coincidentally, methylene chloride replicates segregated with the hexane fraction in one cluster owing to their nonpolar nature.

**Table 1:** Phytoconstituents identified in ethanolic extract and different solvent fractions of *T. alexandrinum* using UPLC-MS/MS

No	RT (min)	Mass (m/z)	Compound name	Class	Formula	Ion type	Fragment ions	Ref
1	0.79	147.01	Citramalic acid	2-hydroxydicarboxylic acid	C_5_H_8_O_5_	[M-H]^−^	103.039	^54^
2	0.79	136.04	Succinic acid	Dicarboxylic acid	C_4_H_6_O_4_	[M + NH4]^+^	n.d	^54^
3	0.82	149.08	2,3,4-Trihydroxy-2-methylbutanoic acid	Hydroxy fatty acids	C_5_H_10_O_5_	[M-H]^−^	131, 113, 105, 87	^55^
4	0.83	197.83	Ascorbic acid	Butenolides	C_6_H_8_O_6_	[M-2H+Na]^−^	131, 121, 117, 101, 85, 73, 61, 43	^54^
5	0.84	195.89	Selenomethionine	Seleno-amino acid	C_5_H_11_NO_2_Se	[M-H]^−^	179, 150, 133, 107, 100	^56^
6	0.89	199.85	Phenylalanine, Amide	Phenylalanine derivative	C_9_H_12_N_2_O	[M+Cl]^−^	146	^56,57^
7	1.06	169.05	Gallic acid	Gallic acids	C_7_H_6_O_5_	[M-H]^−^	125, 97, 79, 69	^58^
8	2.24	175.19	Leucine, Amide	Amino acid amide	C_6_H_14_N_2_O	[M + HCOO-H]^−^	66, 41, 40	^40^
9	2.8	353.28	Caffeoylquinic acid	Quinic acids	C_16_H_18_O_9_	[M-H]^−^	191, 179	^59^
10	3.2	268.16	Adenosine	Purine nucleosides	C_10_H_12_N_4_O_6_	[M + H]^+^	136, 119, 94	^57,60^
11	4.91	113.96	l-Tryptophan	Indolyl carboxylic acids	C_11_H_12_N_2_O_2_	[M + H+Na]^2+^	188, 159, 146, 118	^61^
12	5.14	577.42	Procyanidin b1 or b2	Polyflavonoids	C_30_H_26_O_12_	[M-H]^−^	425, 407, 289, 125	^62^
13	5.35	325.28	Glucose-o-coumaric acid	Hydroxycinnamic acid	C_15_H_18_O_8_	[M-H]^−^	163, 119	^63^
14	6.12	447.23	Luteolin 7-*O*- β-d-glucoside	Flavonoid-o-glycosides	C_21_H_20_O_11_	[M-H]^−^	329, 285	^62,63^
15	6.61	431.23	Genistin	Isoflavonoid-o-glycosides.	C_21_H_20_O_10_	[M-H]^−^	269, 251, 241, 223, 151	^58,64^
16	6.65	447.26	Kaempferol 3-*O*-β-d-Galactopyranoside ; (Trifolianol)	Flavonols-o-glycosides	C_21_H_20_O_11_	[M-H]^−^	327, 285, 284, 255	^63,64^
17	7.69	943.79	Formononetin-7-*O*-glucoside-6′′ -O-acetate	Isoflavonoid-o-glycosides.	C_24_H_24_O_10_	2M-H	267	^58^
18	8.21	285.2	Orobol	Isoflavones	C_15_H_10_O_6_	[M-H]^−^	257, 215, 201, 133	^58^
19	8.69	285.14	Luteolin	Flavones	C_15_H_10_O_6_	[M-H]^−^	257, 241, 217, 199, 175	^65^
20	8.73	299.12	Daidzein	Isoflavones	C_15_H_10_O_4_	[M + HCOO-H]^−^	225, 209, 197	^66^
21	8.74	301.11	3′-Methylorobol	Isoflavones	C_16_H_12_O_6_	[M + H]^+^	273, 245	^58^
22	8.76	301.18	Pratensein	Isoflavones	C_16_H_12_O_6_	[M + H]^+^	286, 153	^61^
23	9.32	285.31	Calycosin	4’-Methoxyisoflavones	C_16_H_12_O_5_	[M-H]^−^	269, 257, 242, 213.1,	^63^
24	9.55	329.35	Genistein	Isoflavones	C_15_H_10_O_5_	[M + CH3COO-H]^−^	251, 241, 227, 151	^58,67^
25	9.69	297.37	4’,5-Dihydroxy-6,7-methylenedioxyisoflavone	Isoflavones	C_16_H_10_O_6_	[M-H]^−^	253	^58^
26	9.69	269.15	7-Hydroxy-4’-methoxyisoflavone (Formononetin)	4’-Methylisoflavones	C_16_H_12_O_4_	[M + H]^+^	253, 237, 225, 213, 137	^61^
27	9.82	327.31	Pseudobaptigenin	Isoflavones	C_16_H_10_O_5_	[M + HCOO-H]^−^	253, 225, 224, 211, 209, 197, 185	^58^
28	10.22	166.1	Cinnamic acid	Cinnamic acids	C_9_H_8_O_2_	[M + NH4]^+^	105	^68^
29	10.74	307.3	Apigenin	Flavones	C_15_H_10_O_5_	[M-2H + K]^−^	225, 201, 153, 151, 149	^38^
30	11.07	941.74	Soyasaponin 1	Triterpene saponins	C_48_H_78_O_18_	[M-H]^−^	795, 615, 457	^69^
31	11.14	941.71	Azukisaponin V (hispidacin)	Triterpene saponins	C_48_H_78_O_18_	[M-H]^−^	795, 633, 457	^58^
32	11.36	283.13	3-Hydroxy-8,9-methylenedioxypterocarpan; ((-)-Maackiain)	Flavones	C_16_H_12_O_5_	[M-H]^−^	173, 149,121, 119	^56^
33	11.28	571.19	Kaempferol	Flavonols	C_15_H_10_O_6_	[2M-H]^−^	255, 227	^70^
34	11.47	315.15	Isorhamnetin	Flavonols	C_16_H_12_O_7_	[M-H]^−^	300, 283, 271, 255, 243, 227, 164, 151	^61^
35	11.93	381.29	Strigol	Strigolactones	C_19_H_22_O_6_	[M+Cl]^−^	363, 284	^61^
36	12.19	113.93	2,3-Dihydroxy-2,4-cyclopentadien-1-one	Vinylogous acids	C_5_H_4_O_3_	[M + H]^+^	83, 67, 65, 55, 41, 39	^58^
37	12.53	279.55	p-Coumaroyl-malic acid	Phenolic acids	C_13_H_12_O_7_	[M-H]^−^	163, 119	^71^
38	12.53	283.13	Prunetin	Isoflavones	C_16_H_12_O_5_	[M-H]^−^	255, 227, 165, 149, 133	^56^
39	13.15	295.31	Naringenin	Flavanones	C_15_H_12_O_5_	[M+Na]^+^	257, 244, 153, 120	^60^
40	13.91	318.39	Chrysoeriol	Flavones	C_16_H_12_O_6_	[M + NH4]^+^	228, 213, 202, 201, 185, 160, 153, 135, 109	^60^
41	14.3	307.3	5,7-Dihydroxy-4’ methoxyisoflavone (Biochanin A)	Isoflavones	C_16_H_12_O_5_	[M+Na]^+^	283, 267, 257, 229	^72^
42	15.19	295.28	Medicagol	Coumestans	C_16_H_8_O_6_	[M-H]^−^	267, 265	^58^
43	15.42	102.06	3,4’,5-Biphenyltriol	Biphenyls	C_12_H_10_O_3_	[M/2 + H]^+^	175, 157, 133, 117, 91, 89, 77	^61^
44	17.06	295.31	Pratenol A	1-benzopyrans	C_14_H_12_O_5_	[M+Cl]^−^	241, 215, 199, 171	^73^
45	17.99	305.3	Coumestrol, 9-Me ether	Coumestan	C_16_H_10_O_5_	[M+Na]^+^	283, 267, 255, 239, 147	^72^
46	19.1	279.26	2’,4,4’-Trihydroxychalcone	Chalcones	C_15_H_12_O_4_	[M+Na]^+^	135, 121, 93	^74^
47	19.15	301.27	2,4’,5,7-Tetrahydroxyisoflavanone, 4’-Me ether	Isoflavanones	C_16_H_14_O_6_	[M-H]^−^	283, 253, 241, 149, 135, 105, 77	^67^
48	19.17	309.32	Phloretin	Dihydrochalcone	C_15_H_14_O_5_	[M+Cl]^−^	255, 167, 125	^67^
49	19.31	271.32	2’,4’,7-Trihydroxyisoflavan, 4’-Me ether	Isoflavanones	C_16_H_16_O_4_	[M-H]^−^	255, 239, 227, 225, 135	^75^
50	19.44	271.32	1-(2,4-Dihydroxyphenyl)-2-(4-hydroxyphenyl)ethanedione; 4’-Me ether	Stilbenes	C_15_H_12_O_5_	[M-H]^−^	135, 119, 109	^76^
51	20.47	313.38	Linolenic acid	Polyunsaturated fatty acid	C_18_H_30_O_2_	[M+Cl]^−^	259, 233	^75^
52	22.05	313.35	3,6a,8,9-Tetrahydroxypterocarpan; 8,9-Methylene, 3-Me ether	Pterocarpan	C_17_H_14_O_6_	[M-H]^−^	297, 283, 269	^77^
53	22.21	257.27	1-Octen-3-ol	Fatty alcohols	C_8_H_16_O	[2 M+Na]^+^	111, 83, 71, 57, 55	^68^
54	22.25	239.24	Bergaptol; Me ether (Bergapten)	5-hydroxypsoralens	C_12_H_8_O_4_	[M+Na]^+^	202, 189, 173, 145	^67^
55	24.82	767.45	3,24-Dihydroxy-12-oleanen-29-oic acid; 3β-form, 3-O-[α-L-Arabinopyranosyl-(1 → 2)-β-d-glucuronopyranoside	Triterpenoids	C_41_H_66_O_13_	[M + H]^+^	749, 721, 635, 455, 437	^75^
56	25.23	309.35	2’,4’,7-Trihydroxyisoflavan, 2’,7-Di-Me ether	Isoflavan	C_17_H_18_O_4_	[M+Na]^+^	269, 255, 161	^78^
57	31.09	311.18	5,16-Dimethyl-3-methylene-1,2-heptadecanediol	long-chain fatty alcohols	C_20_H_40_O_2_	[M-H]^−^	293, 279, 251, 59	^79^

### Network pharmacology analysis of *T. alexandrinum* metabolites

**Fig. 3:**
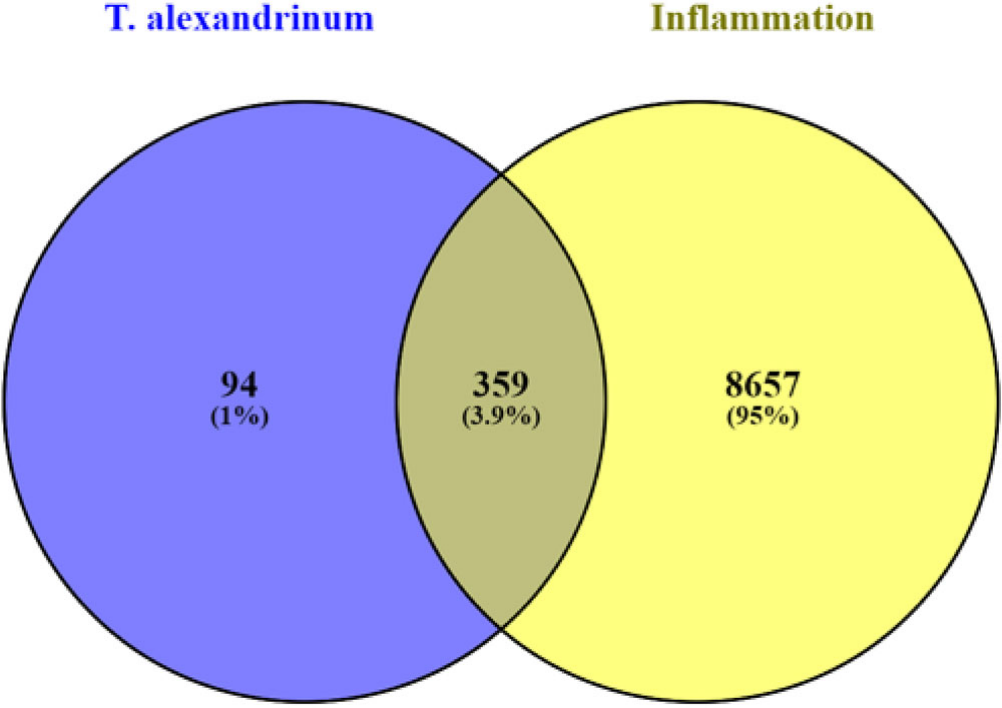
Venn diagram illustrating the number of common genes shared between *T. alexandrinum*-related genes and inflammatory genes.

To analyze the underlying anti-inflammatory mechanisms of annotated compounds in each *T. alexandrinum* solvent fraction, interactions with the linked targets and identification of involved pathways were investigated using “network pharmacology” analysis^16^. The interactions between endogenous metabolites of *T. alexandrinum* and proteins associated with inflammation were elucidated by constructing a compound-target (C-T) network. Initially, 453 protein targets were associated with the 57 compounds identified through UPLC-MS/MS analysis as evolved from STITCH 5.0, SEA, and Swiss target prediction databases. Venn diagram (Fig. 3) revealed the presence of 359 common genes between “inflammation-related targets” and “the prospective targets” of *T. alexandrinum* endogenous metabolites. The “combined score” serves as a metric for assessing the strength of interactions between the compounds and the genes; compounds with higher combined scores indicate robust and precise interactions with their corresponding genes (Table S1).

**Fig. 4:**
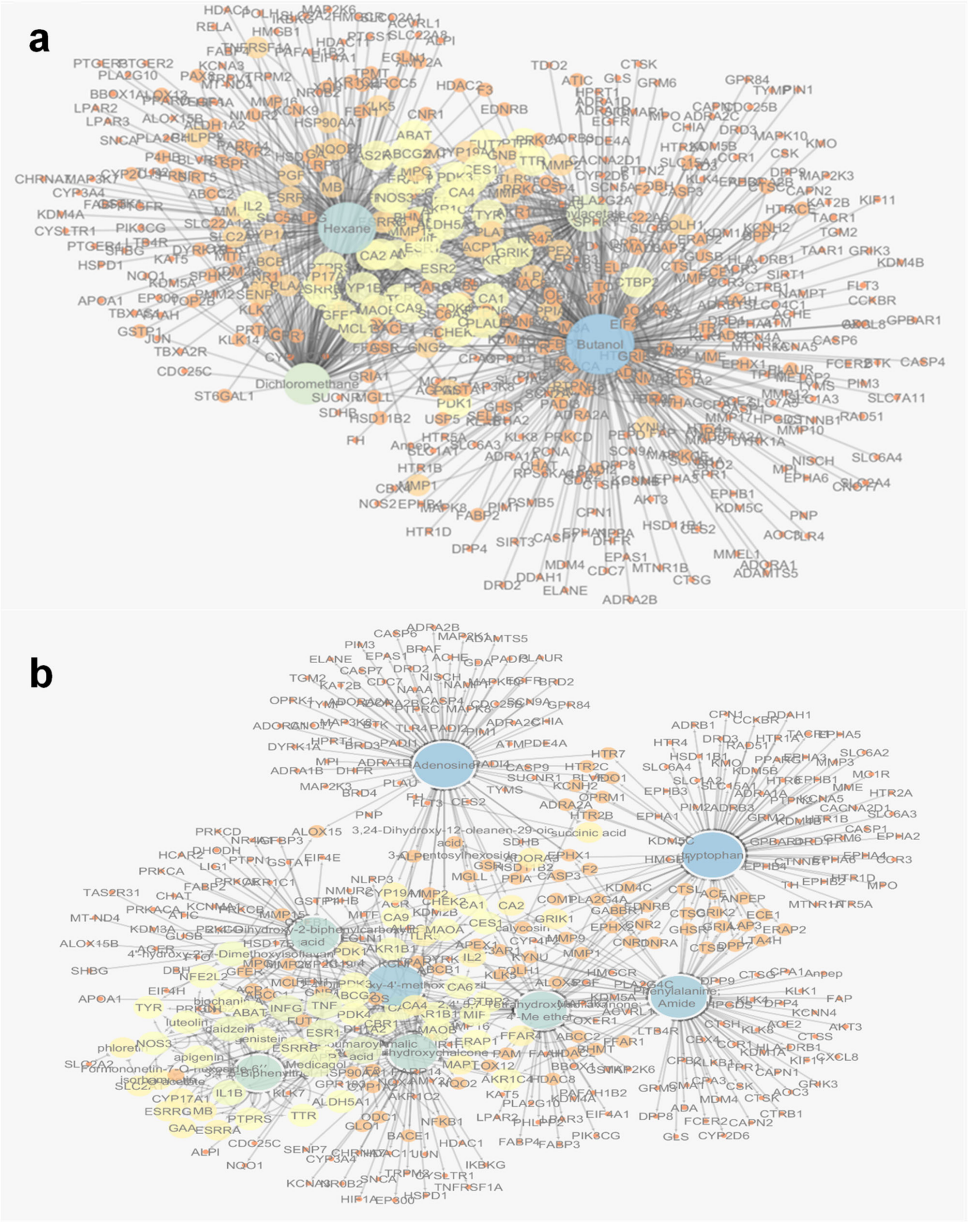
Networks of *T. alexandrinum* with inflammatory genes. Fraction-target (**a**) and compound–target (C–T) (**b**) interactions (increase in node size and color gradient from yellow to blue indicates higher degree of interaction).

The fraction-target network constructed (Fig. 4a) consisted of 478 nodes (four solvent fractions and 474 target genes) and 955 edges. It was evident from the node size and color that the butanol fraction scored the highest interactions. This was confirmed by topological parameters of the fraction-target network (Table S2) examined using the network analyzer built into Cytoscape 3.10.2.

Additionally, the compound-target (C-T) network constructed (Fig. 4b) highlighted the multi-target nature of *T. alexandrinum* phytoconstituents. Analysis of the interaction data (Fig. 4b) and topological parameters (Table 2) of active constituents revealed that tryptophan exhibited the highest interaction percentage, followed by adenosine, 2,4-dihydroxy-4’-methoxybenzil, and phenylalanine amide. By referring to literature, tryptophan, the top-scored compound, posed as a potential regulator of an ongoing inflammatory process and, therefore, a potential therapeutic, non-chemical strategy. This was evident from investigating the molecular patterns of anti-inflammatory processes that was favored by tryptophan when fed to European seabass injected intraperitonially with Freund’s Incomplete Adjuvant (FIA) for induction of inflammation. Surprisingly, gene expression of transforming growth factor β, interleukin-10, macrophage colony-stimulating factor receptor, and interleukin 34 was significantly suppressed by dietary tryptophan^17^. Pinpointing adenosine, the second top evolved hit, it acts as a strong anti-inflammatory by regulating the activities of different immune cells, such as neutrophils, macrophages, monocytes, dendritic cells, and lymphocytes, especially in conditions where the immune system is hyperactive^18^. Interestingly, the erythema and consequent heat loss linked to inflammation are therefore probably caused by the release of adenosine at inflammatory sites. It’s interesting to note that decreased adenosine production causes dramatic vascular leakage because it inhibits the activation of adenosine A2B receptors on the vascular endothelium. This suggests that adenosine released at inflammatory sites reduces the noticeable swelling^19^.

**Table 2:** *T. alexandrinum* main active constituents network node topological parameters

Compound name	Betweenness centrality	Closeness centrality	Degree
Tryptophan	0.30201774	0.38150289	90
Adenosine	0.32129104	0.39442231	87
2,4-Dihydroxy-4’-methoxybenzil	0.21672042	0.38521401	77
Phenylalanine amide	0.25310615	0.37714286	77
2,4’,5,7-Tetrahydroxyisoflavanone; 4’-Me ether	0.1836031	0.375	59
2’,4’-Dihydroxy-2-biphenylcarboxylic acid	0.17169462	0.37429112	56
2’,4,4’-Trihydroxychalcone	0.14563808	0.37358491	55
3,4’,5-Biphenyltriol	0.0330403	0.31781701	51
p-Coumaroyl-malic acid	0.01808993	0.34859155	22
Medicagol	0.02741832	0.34859155	17
4’-Hydroxy-2’,7-dimethoxyisoflavan	0.02443693	0.28862974	13
Genistein	0.00186708	0.28695652	8
Apigenin	3.41E-04	0.28654124	6
Luteolin	0.00113104	0.2832618	6
Biochanin A	0.00582209	0.28407461	5
Isorhamnetin	6.31E-04	0.25647668	3
Phloretin	0.00602472	0.2578125	3
Succinic acid	1	1	3
3,24-Dihydroxy-12-oleanen-29-oic acid; 3-*O*-pentosylhexoside	0	1	2
Calycosin	1	1	2
Formononetin-7-*O*-hexoside-6′′ -O-acetate	5.20E-05	0.25614489	2

On the other side, examination of the targeted genes (Fig. 4b) and (Table 3) indicated that the genes; interferon-γ (IFNG), tumor necrosis factor- α (TNF-α), interleukin-6 (IL-6), interleukin-1β (IL1B), and estrogen sensitive receptor-α (ESR1) were the most significantly enriched, showing the highest degrees of interaction with *T. alexandrinum* compounds in the C-T network, suggesting their potential pivotal role in inflammation suppression.

**Table 3:** *T. alexandrinum* main active ingredient’s targets, network node topological parameters

Gene name	Betweenness centrality	Closeness centrality	Degree	Gene name	Betweenness centrality	Closeness centrality	Degree
IFNG	0.038335	0.345852	17	C3AR1	0.002631	0.300227	2
TNF	0.063694	0.390148	17	CASP3	0.007829	0.315538	2
IL-6	0.028745	0.341674	14	CNR1	0.004844	0.308171	2
IL1B	0.005337	0.28105	13	CNR2	0.004844	0.308171	2
ESR1	0.032593	0.371831	9	COMT	0.009704	0.317054	2
AKR1B10	0.017185	0.345248	7	CTSB	0.003498	0.306739	2
NOS3	1.23E-04	0.224363	7	CTSC	0.003498	0.306739	2
ABCG2	0.008053	0.315538	6	CTSL	0.003498	0.306739	2
APP	0.002113	0.296629	6	CYP1A2	6.57E-04	0.292251	2
CA6	0.022659	0.366327	6	DPP7	0.003498	0.306739	2
NFE2L2	0.002113	0.296629	6	ECE1	0.003498	0.306739	2
ABAT	0.001752	0.295743	5	EDNRA	0.004844	0.308171	2
AKR1B1	0.020977	0.352627	5	EDNRB	0.004844	0.308171	2
ALDH5A1	0.001752	0.295743	5	EPHX1	0.007829	0.315538	2
CBR1	0.005785	0.317562	5	EPHX2	0.004844	0.308171	2
CES1	0.038161	0.397192	5	ERAP2	0.003498	0.306739	2
ESRRB	0.002071	0.285097	5	F2	0.007829	0.315538	2
MAOB	0.005731	0.316547	5	F3	3.44E-05	0.273292	2
MIF	0.030437	0.340499	5	FFAR1	0.004071	0.302984	2
TTR	6.31E-04	0.27673	5	FOLH1	0.002631	0.300227	2
AKR1C4	0.008347	0.323794	4	FOS	3.44E-05	0.273292	2
CA1	0.025531	0.37044	4	FUT7	0.001196	0.295302	2
CA9	0.0186	0.351375	4	GABBR1	0.004844	0.308171	2
CTBP2	0.008292	0.330275	4	GFER	0.001196	0.295302	2
ERAP1	0.005687	0.311566	4	GHSR	0.003498	0.306739	2
MAOA	0.017864	0.352	4	GLO1	3.44E-05	0.273292	2
PDK3	0.007822	0.315036	4	GNB1	2.93E-04	0.275958	2
PDK4	0.007822	0.315036	4	GNG2	2.93E-04	0.275958	2
PTPRS	2.12E-04	0.263473	4	GRIA1	0.003498	0.306739	2
TYR	2.59E-04	0.244595	4	GRIK2	0.003498	0.306739	2
ADORA3	0.021185	0.357724	3	GSR	0.005359	0.307692	2
CA2	0.015041	0.347064	3	HDAC2	0.003554	0.30206	2
CA4	0.004076	0.310102	3	HDAC8	0.003554	0.30206	2
CHEK2	0.021185	0.357724	3	HSD11B2	0	1	2
CYP17A1	0	0.241316	3	HSP90AA1	6.57E-04	0.292251	2
CYP19A1	0.009052	0.309617	3	HTR2B	0.007202	0.320648	2
ESRRA	0	0.241316	3	HTR2C	0.007202	0.320648	2
ESRRG	0	0.241316	3	HTR7	0.007202	0.320648	2
FFAR4	0.008582	0.328631	3	IDO1	0.007202	0.320648	2
GAA	0	0.241316	3	KCNH2	0.007202	0.320648	2
GRIK1	0.019715	0.339332	3	KDM4C	0.004844	0.308171	2
IL2	0.001578	0.297074	3	KLK5	0.002933	0.306739	2
MAPT	8.74E-04	0.293116	3	KYNU	0.002631	0.300227	2
MB	0	0.241316	3	LAP3	0.003498	0.306739	2
PDK1	0.002986	0.309617	3	LPAR1	0.001623	0.291391	2
SLC2A1	9.82E-05	0.224109	3	LTA4H	0.003498	0.306739	2
TLR9	0.006502	0.308171	3	MCL1	0.001196	0.295302	2
ABCB1	0.001578	0.297074	2	MGLL	0.005359	0.307692	2
ABCC1	0.001047	0.279859	2	MMP1	0.009146	0.309617	2
ABCC2	0	0.272915	2	MMP15	0.00117	0.281851	2
ACE	0.003498	0.306739	2	MMP2	0.004672	0.306739	2
ACP1	2.93E-04	0.275958	2	MMP26	0.00117	0.281851	2
ADRA2A	0.007202	0.320648	2	MMP9	0.009146	0.309617	2
ALOX12	0	0.272915	2	MPG	2.93E-04	0.275958	2
ALOX15	0.00621	0.289262	2	NFKB1	3.44E-05	0.273292	2
ALOX5	0.002933	0.306739	2	NQO2	6.57E-04	0.292251	2
ALPL	0.004279	0.312549	2	ODC1	3.44E-05	0.273292	2
ANPEP	0.003498	0.306739	2	OPRM1	0.007202	0.320648	2
APEX1	0.002631	0.300227	2	PAM	3.97E-04	0.279464	2
BACE1	3.44E-05	0.273292	2	PLA2G4A	0.004844	0.308171	2
BHMT	0.004071	0.302984	2	PPIA	0.013586	0.310588	2

A pleiotropic cytokine, interferon-γ, revealed as the most inflected gene by *T. alexandrinum* constituents, has a variety of impacts on both innate and adaptive immunity as well as the inflammatory response^20^. A number of potentially lethal hyperinflammatory or immune-mediated illnesses are caused by an excess of INF-γ. It has been implicated in a number of hyperinflammatory diseases, including primary hemophagocytic lymphohistiocytosis, different types of secondary hemophagocytic lymphohistiocytosis, including macrophage activation syndrome, and cytokine release syndrome, according to data from animal models and/or translational studies in patients^21^. Another cytokine that affects different cell types pleiotropically is tumor necrosis factor-alpha (TNF-α), the second top hit in our network. It is known to play a role in the pathophysiology of some inflammatory and autoimmune illnesses and has been recognized as a key regulator of inflammatory responses. TNF-α is a homotrimer protein that is primarily produced by natural killer cells, T-lymphocytes, and activated macrophages. It is functionally known to activate a number of different inflammatory chemicals, such as chemokines and other cytokines^22^. A few large protein monoclonal antibodies have been approved by the FDA as TNF Inhibitors for the treatment of rheumatoid arthritis. Several natural products have been reported to inhibit TNF either in vitro or by computational docking studies, for example, ginseng saponins and ergostane derivatives^23^.

Thirdly, interleukin-6 (IL-6) is typically characterized as a pro-inflammatory cytokine with pleiotropic effects on both inflammation and immune response. Upon activation of pattern recognition receptors, leukocytes and stromal cells release IL-6 as part of the innate immune response. The B and T cell response is then triggered by IL-6, which also attracts immune cells. Its dysregulation plays a pathological effect on chronic inflammation and autoimmunity. This cytokine is involved in the progress of many autoimmune diseases for instance, rheumatoid arthritis, systemic lupus erythematosus, inflammatory myopathies, and others^24^. IL-6 receptor blockers have been developed as promising therapeutic targets for preventing or treating immune-mediated diseases. Among these, tocilizumab, an approved humanized anti-IL-6 receptor antibody used as monotherapy for the treatment of rheumatoid arthritis^25^.

Furthermore, in the healthy organism, IL-1β plays a key role in homeostatic processes like feeding, sleep, and temperature regulation. However, its overproduction is associated with pathophysiological changes observed in various diseases, including rheumatoid arthritis, neuropathic pain, inflammatory bowel disease, osteoarthritis, vascular disease, multiple sclerosis, and Alzheimer’s disease^26^. Additionally, IL-1β is implicated in chronic conditions such as stroke, myocardial infarction, and type 2 diabetes^27^. Research has further linked IL-1β to the production of carcinogenic mediators, breast cancer progression, and the development of bone metastases in breast cancer patients^23^. Consequently, targeting IL-1β presents a promising therapeutic strategy for numerous diseases, including breast cancer metastasis^28,29^.

Eventually, estrogen sensitive receptor-α (ESR1) controls a number of intricate physiological functions in humans. Numerous conditions, such as inflammations, endometriosis, breast, ovarian, and prostate cancer, abnormalities of the bones, lung cancer, and cardiovascular diseases can be brought on by abnormal ESR1 signaling^30^.

**Fig. 5:**
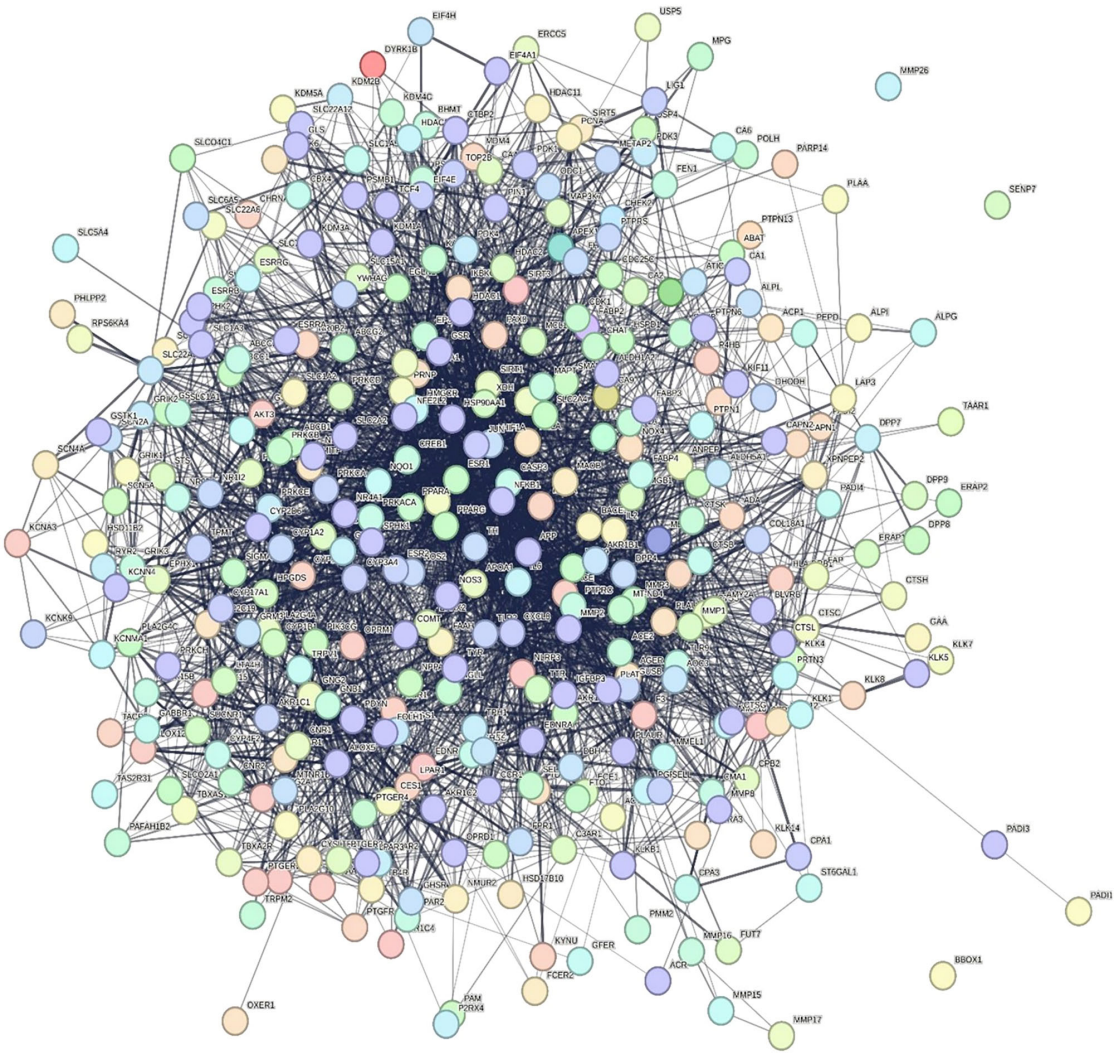
Protein–protein interaction diagram of *T. alexandrinum* target genes related to inflammation.

Additionally, protein–protein interactions were assessed using the STRING database and visualized through a protein–protein (P–P) network analysis. This network revealed strong correlations among the identified potential anti-inflammatory target proteins, implying possible regulatory interactions between them (Fig. 5).

To explore potential metabolic pathways associated with inflammation, the target genes were submitted to Kyoto Encyclopedia of Genes and Genomes (KEGG) pathway analysis, with annotations limited to Homo sapiens. As illustrated in (Fig. S2 and Table S3), the target genes were implicated in 66 inflammation-related pathways (with *P* values <0.001). The most enriched pathways included the arachidonic acid metabolism pathway, followed by the fluid shear stress and atherosclerosis pathway, hepatitis B pathway, microRNAs in cancer, glutamatergic synapse, apoptosis, HIF-1 signaling pathway, and insulin resistance pathway. The constructed networks were integrated to form a fraction-compound–target–pathway network (Fig. S3), indicating strong interrelations among the studied compounds, inflammation-related targets, and associated pathways.

The arachidonic acid (AA) metabolism pathway, the top-listed pathway in network, plays a pivotal role in inflammation. After irritation or damage, enzyme systems release and oxygenate arachidonic acid, which results in the production of eicosanoids, a significant class of inflammatory mediators. Eicosanoid release is now understood to be essential to the inflammatory process. For instance, the cyclooxygenase enzyme pathway produces prostaglandins and other prostanoids, which have strong inflammatory effects. Prostaglandin E2 is easily found in acute inflammatory exudates from equine sources^31^. AA-derived mediators, especially lipoxins and epoxyeicosatrienoic acids, have anti-inflammatory properties. They can suppress the expression and secretion of pro-inflammatory cytokines such as TNF-α, IL-1β, IL-6, and IFN-γ by modulating signaling pathways in immune cells^32^. For instance, epoxyeicosatrienoic acids have been shown to inhibit TNF-α-induced apoptosis and inflammation in endothelial cells, partly by upregulating autophagy and reducing caspase activation^33^. Secondly, Variations in blood flow patterns within arteries can change the adaptive characteristics of vascular endothelial cells, influencing their functions and contributing to early atherosclerosis lesions. Atherosclerotic plaques are often located at curved or bifurcated arteries, where oscillating shear stress predominates. Oscillating shear stress can trigger endothelial cells to adopt pro-inflammatory traits, leading to increased inflammation, oxidative stress, mitochondrial dysfunction, metabolic issues, and heightened endothelial permeability, all of which facilitate atherosclerosis progression. In contrast, straight arteries experience stable laminar shear stress, which encourages endothelial cells to adopt an anti-inflammatory phenotype, enhancing their function and helping to slow down atherosclerosis^34^. Moreover, laminar or steady shear stress promotes an atheroprotective, anti-inflammatory endothelial phenotype. It induces gene and protein expression that suppresses inflammation and inhibits the production of pro-inflammatory cytokines such as TNF-α, IL-1β, IL-6, and IFN-γ^35,36^. Lastly, hepatitis B virus x protein (HBx) plays a significant role in enhancing signal transduction related to innate immunity and inflammation during HBV infection. HBx activates toll-like receptor (TLR) and nuclear factor-kappaB (NF-κB) signaling pathways, leading to increased expression of pro-inflammatory cytokines. Additionally, HBx stimulates the activation of the NOD-like receptor protein 3 inflammasome, which accelerates the release of IL-1β and IL-18^37^. In addition, the hepatitis B inhibition pathway mechanistically connects with the suppression of TNF-α, IL-1β, IL-6, and IFN-γ by reducing viral replication and antigenic stimulation, promoting regulatory immune responses, and directly modulating cytokine production through both immune and therapeutic mechanisms^38,39^.

### Isolation of bioactive compounds from *T. alexandrinum* metabolites

Network pharmacology analysis illustrated the potential of chemical compounds in the butanol fraction to be effective against different inflammatory targets (Fig. 4b and Table 2). Hence, it was chosen for further purification to isolate compounds for investigating their anti-inflammatory potential in vitro. Two compounds were isolated, and their structures were confirmed through comparing their spectral data, ^1^H, ^13^C NMR, and HRESI-MS spectra to those in previous literature^4,40–42^ (Figs. S4–S9 and supplementary file).

Compound 1 Tryptophan: ^1^H-NMR (400 MHz, DMSO, d6) *δ* ; 11.02 (s, 1H, NH1), 7.58 (d, *J* = 7.8 Hz, 1H, H-4), 7.36 (dt, *J* = 8.2, 1.0 Hz, 1H, H-7), 7.24 (d, *J* = 2.3 Hz, 1H, H-2), 7.07 (ddd, *J* = 8.1, 6.9, 1.2 Hz, 1H, H-6), 6.98 (ddd, *J* = 8.0, 7.0, 1.1 Hz, 1H, H-5), 3.50 (dd, *J* = 8.7, 4.1 Hz, 1H, H-8a), 3.33 (dd, *J* = 15.3, 4.1 Hz, 1H, H-8b), 3.00 (dd, *J* = 15.1, 8.8 Hz, 1H, H-9). ^13^C NMR (100 MHz, DMSO, d6) *δ*; 170.83 (C-10), 136.82 (C-7a), 127.76 (C-3a), 124.60 (C-2), 121.33 (C-6), 118.86 (C-5), 118.72 (C-4), 111.81 (C-7), 110.01(C-3), 55.22 (C-9), 27.58 (C-8). HRESI-MS spectra of compound 1 showed a molecular ion peak [M + H]^+^ at *m/z* 205.098 (calculated for C_11_H_13_N_2_O_2_, 205.0977).

Compound 2 Adenosine: ^1^H-NMR (400 MHz, DMSO, d6) *δ* ; 8.35 (s, 1H, H-8), 8.14 (s, 1H, H-2), 5.89 (d, *J* = 6.2 Hz, 1H, H-1’), 4.69–4.55 (m, 1H, H-2’), 4.15 (dd, m, 1H, H-3’), 3.97 (q, *J* = 3.4 Hz, 1H, H-4’), 3.68 (dd, *J* = 12.1, 3.6 Hz, 1H, H-5’a), 3.56 (dd, *J* = 12.1, 3.6 Hz, 1H, H-5’b). ^13^C NMR (100 MHz, DMSO, d6) *δ*; 156.64 (C-6), 152.85 (C-2), 149.53 (C-4), 140.40 (C-8), 119.83 (C-5), 88.39 (C-1’), 86.37 (C-4’), 73.91 (C-2’), 71.13 (C-3’), 62.15 (C-5’). HRESI-MS spectra of compound 2 showed a molecular ion peak [M + H]^+^ at *m/z* 268.104 (calculated for C_10_H_14_N_5_O_4_, 268.1045).

### In vitro cell viability assay on WI38 human fibroblast cells

Network pharmacology analysis revealed the anti-inflammatory activity of *T. alexandrinum* butanol fractions and their compounds, targeting various inflammatory cytokines and pathways (Table 2 and S3). This was assessed using WI38 human fibroblast cell model to examine the mechanism underlying the anti-inflammatory effects of the total extract and solvent fractions of *T. alexandrinum*.

**Fig. 6:**
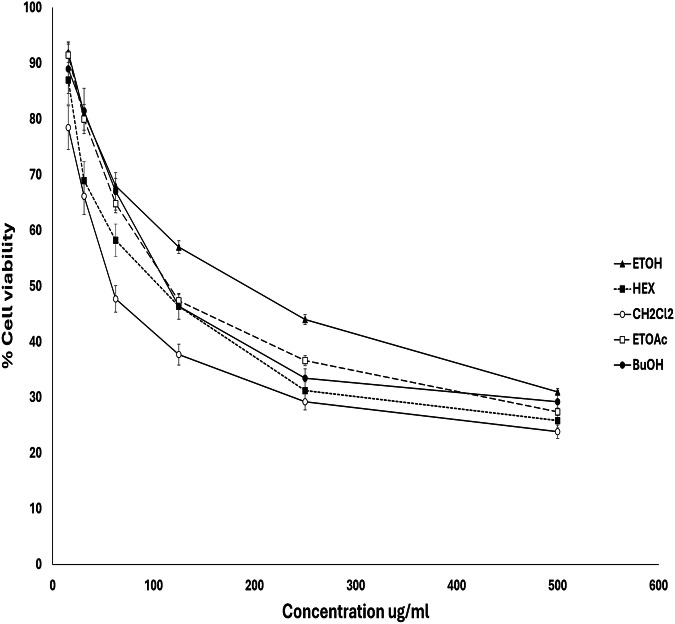
The effect of *T. alexandrinum* extract and fractions on the viability of WI38 human fibroblast cells using the MTT assay. The data are represented as the mean ± standard deviation (SD) of triplicate experiments.

The first cell viability assay was performed using an MTT in vitro assay. The results showed that the plant total extract along with its ethyl acetate, butanol and hexane fractions were non cytotoxic to WI38 cells (Fig. 6). They exhibited IC50 values of 181, 144.4, and 136.9 μg/ mL, respectively which were superior to that of the anti-inflammatory drug, piroxicam that had IC50 of 100 μg/mL. On the contrary, the methylene chloride fraction showed a low IC50 value of 71 μg/ mL, indicating that compounds in the less polar fractions of *T. alexandrinum* may exert cytotoxic effects on WI38 cells.

### Effect of *T. alexandrinum* total extract, solvent fractions, and isolated compounds on LPS-induced pro-inflammatory cytokines in WI38 cells

Inflammatory triggers, such as immune-stimulatory lipopolysaccharides (LPS), are well-known to enhance the release of various pro-inflammatory mediators, including TNF-α, IL-1β, IL-6, and INF-γ^43^. These cytokines were assessed in our study to evaluate the anti-inflammatory actions of *T. alexandrinum*. Levels of TNF-α, IL-1β, IL-6, and INF-γ were measured in the culture supernatant of LPS-simulated WI38 cells, following treatment with the total extract, solvent fractions, and hit compounds identified through network pharmacology. The anti-inflammatory potential was evaluated in comparison to the synthetic agent piroxicam.

**Fig. 7:**
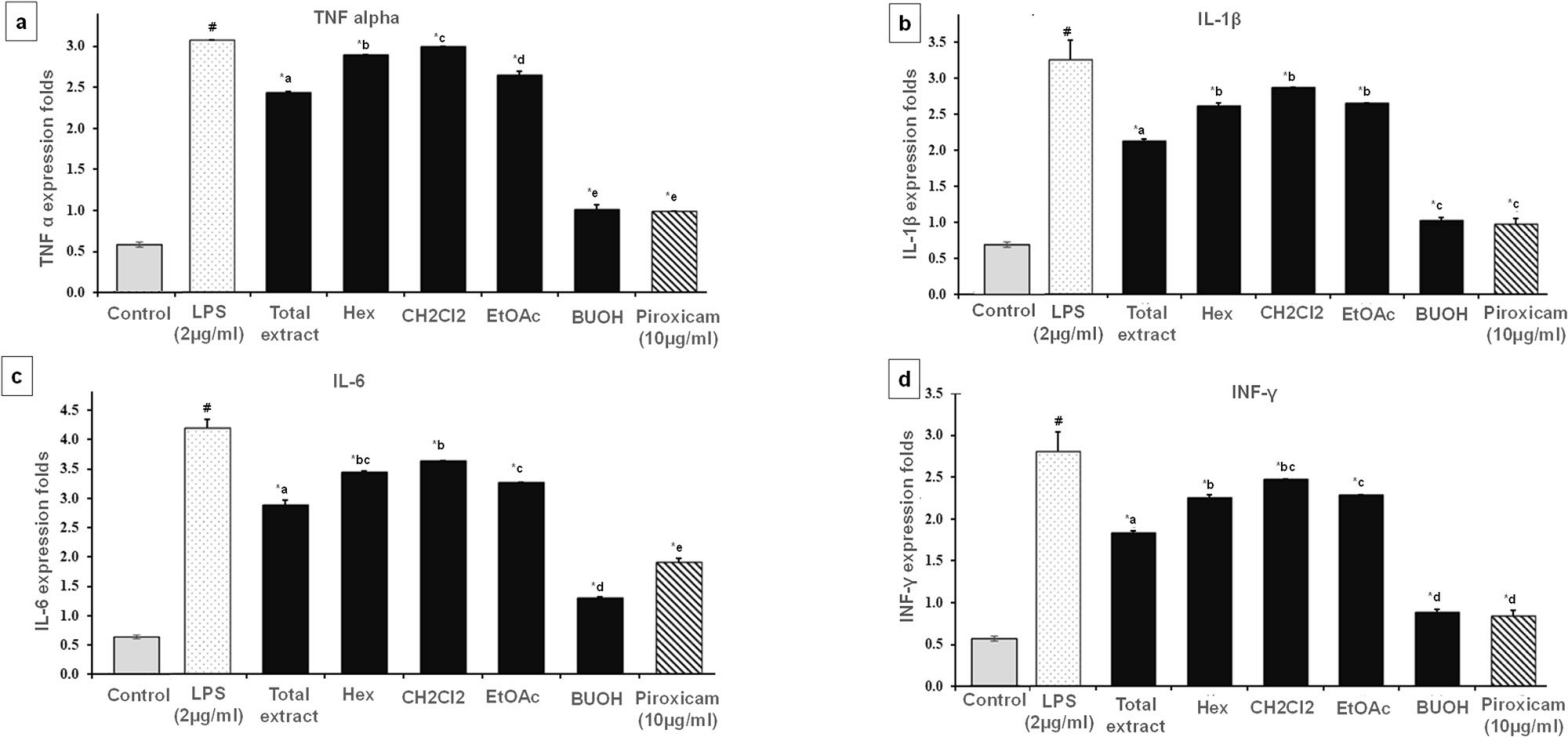
The effect of *T. alexandrinum* total extract and solvent fractions on LPS-induced cytokines production in WI38 cells. Gene expression levels of **a** TNF-α, **b** IL-1 β, **c** IL-6, and **d** INF-γ measured by real-time polymerase chain reaction (PCR). Data were expressed as the mean ± SD (*n* = 3). ^#^*p* < 0.001, indicates statistically significant difference compared to the control group, *p < 0.001 compared to the LPS group, means without a common letter are significantly different (*p* < 0.05).

**Fig. 8:**
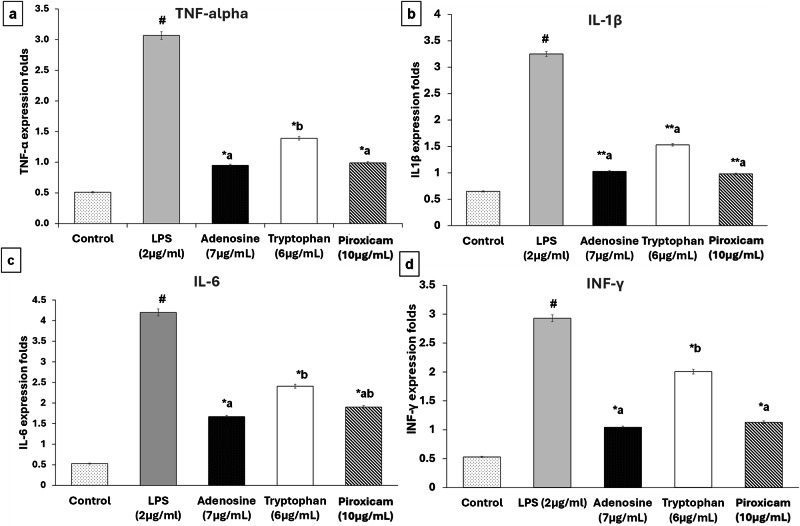
The effect of *T. alexandrinum* isolated compounds on LPS-induced cytokine production in WI38 cells. Gene expression levels of **a** TNF-α, **b** IL-1β, **c** IL-6, and **d** INF-γ measured by real-time polymerase chain reaction (PCR). Data were expressed as the mean ± SD (*n* = 3). ^#^*p* < 0.001, indicates statistically significant difference compared to the control group, *p < 0.001, **p < 0.01 compared to LPS group, means without a common letter are significantly different (*p* < 0.05).

As illustrated in Figs. 7, 8, LPS efficiently induced the production of all four pro-inflammatory mediators. Pretreatment with the total extract, solvent fractions, and isolated compounds of *T. alexandrinum* significantly suppressed the LPS-induced expression of TNF-α, IL-1β, IL-6, and INF-γ. Among the plant solvent fractions, the butanol fraction exhibited the most potent anti-inflammatory effects, reducing cytokine levels to levels comparable to piroxicam (Fig. 7). Furthermore, the compounds adenosine and tryptophan isolated from the butanol fraction markedly inhibited inflammatory cytokine expression relative to the LPS group (Fig. 8).

The increase in TNF-α levels were reduced by 1.01-, 0.946-, and 1.3885-fold following treatment with butanol, adenosine, and tryptophan, respectively. Similarly, IL-1β expression was attenuated by 1.02-, 1.03-, and 1.5295-fold for butanol, adenosine, and tryptophan, respectively. The butanol fraction and its active compounds also showed superior downregulation of IL-6 and INF-γ compared to piroxicam (Fig. 8). For INF-γ, pretreatment with butanol, adenosine, and tryptophan resulted in 1.05-, 1.039-, and 2.005-fold decreases, respectively, whereas piroxicam achieved a 1.13-fold reduction. Among the induced inflammatory mediators, the cytokine IL-6 exhibited the most pronounced downregulation in response to *T. alexandrinum*. The butanol fraction, adenosine, and tryptophan caused 1.3-, 1.67-, and 2.41-fold decreases in IL-6 levels, respectively, compared to the 1.9-fold reduction observed with piroxicam (Figs. 7, 8 and Table S4).

Taken together, the in vitro anti-inflammatory assay using the LPS-induced WI38 cell model revealed the strong anti-inflammatory properties of *T. alexandrinum* total extract, and particularly its butanol fraction. These findings align with the network pharmacology analysis, which highlighted the effectiveness of compounds in the most polar fraction of the plant extract. Moreover, tryptophan and adenosine, identified as top-scoring compounds in the network pharmacology study due to their strong interactions with inflammation-related genes, were confirmed to exhibit potent anti-inflammatory effects in the in vitro cell model, with adenosine showing the strongest effect. This came in agreement with previous studies have demonstrated the anti-inflammatory effects of a number of *Trifolium* species^44^. For instance, the leaf extract of the well-known red clover, *Trifolium pratense*, was found to potentially inhibit the induction of many inflammatory markers as prostaglandin E2 (PGE2), cyclooxygenase-2 (COX-2) and the cytokines TNF-α, IL-1β, and IL-6 in LPS-induced cells^45^. Lee et al.^4^ highlighted the anti-inflammatory efficacy of anthocyanins in red clover, targeting monocyte chemoattractant protein (MCP)1, in addition to COX-2, TNF-α, and IL-1. In-vivo studies have also shown the anti-inflammatory properties of *T. riograndense* and *T. resupinatum* var. *microcephalum* in rat paw edema and arthritic rat models, respectively^44,45^.

Additionally, most studies attribute the anti-inflammatory properties of *Trifolium* species primarily to their isoflavone content. Anthocyanins have also been identified as another class of compounds with reported anti-inflammatory activity^44,45^. In our study, the anti-inflammatory activities of *T. alexandrinum* were strongly linked to nitrogen-containing compounds, the purine nucleoside adenosine, and the amino acid tryptophan. As a functional food, clovers (*Trifolium* species) are among the important sources of sprouts rich in phytoestrogens (isoflavones) and offer notable nutritional value. This study highlights *T. alexandrinum* as a promising candidate for further research into its potential use as a functional food with anti-inflammatory properties^46^.

In conclusion, this study investigates the anti-inflammatory properties of berseem clover, *T. alexandrinum*, identified through network pharmacology analysis and confirmed via in vitro testing. The anti-inflammatory activity is strongly associated with the types of constituents, with polar compounds and fractions, especially butanol, showing the most significant effects in reducing expression levels of TNF-α, IL-1β, IL-6, and INF-γ in an LPS-induced WI38 cell model. Notably, the purine nucleoside adenosine and the amino acid tryptophan exhibited promising anti-inflammatory effects comparable to the therapeutic agent piroxicam. These findings provide valuable insights into the therapeutic potential of *T. alexandrinum* and its constituents, suggesting future in vivo and clinical investigations to develop anti-inflammatory treatments. Furthermore, they emphasize the potential of *T. alexandrinum* as a functional food ingredient, valued for both its nutritional and therapeutic properties.

## Methods

### Plant material and extract preparation

The aerial parts of *T. alexandrinum* were collected from Alexandria, Egypt, in December 2023. Identification of the plant material was confirmed by Professor Dr. Selim Zidan Heneidy, professor of Applied Ecology, Faculty of Science, Alexandria University. Voucher specimen (TA108) has been deposited in the herbarium of the Pharmacognosy Department, Faculty of Pharmacy, Alexandria University. The dried powdered aerial parts 90 g were soaked in 70% ethanol and concentrated under reduced pressure. Dried ethanolic extract was re-dissolved in 90% ethanol and fractionated using the Kupchan partitioning method^47^, with hexane, followed by methylene chloride then ethyl acetate, and finally *n*-butanol to yield 39, 20, 12.5, and 9 g dry fractions, respectively. Dried extracts and fractions were used at various concentrations for in vitro cell experiments.

### UPLC- ESI MS/MS conditions

Samples of *T. alexandrinum* extract and solvent fractions were analyzed in triplicate (H1–H3 for hexane, D1–D3 for dichloromethane, E1–E3 for ethyl acetate and B1–B3 for butanol fractions) using an UPLC XEVO TQD triple quadruple instrument (Waters Corporation, Milford, MA01757 USA) in positive and negative ion modes. The UPLC system includes a Waters Acquity QSM pump, a LC-2040 (Waters) autosampler, degasser, and Waters Acquity CM detector. A Waters Acquity UPLC BEH C18 column (50 mm × 2.1 mm ID × 1.7-μm particle size) was used for the chromatographic separation at 30 °C and a flow rate of 0.2 ml/min. The mobile phase consisted of ultrapure water + 0.1% formic acid (Phase A) and acetonitrile + 0.1% formic acid (Phase B). gradient elution adopted was as follows: 0.0–2.0 min, 10% B; 2.0–5.0 min, 30% B; 5.0–15.0 min, 70% B; 22.0 min, 90% B; 22.0–25.0 min, 90% B; 26.0 min, 100% B; 26.0-29.0 min, 100% B; 32.0 min, 10% B.

ESI MS/MS analyses were performed using a triple quadrupole (TQD) mass spectrometer in conjunction with an electrospray ionization (ESI) source in the positive and negative ionization modes. The optimized ESI operating conditions were as follows: capillary voltage of 3 kV, cone voltage; 35 V, the ion source temperature was 150 ^◦^C, the nebulizer (nitrogen gas) pressure was 35 psi, drying and sheath gas (N2) temperature was 440 and 350 ^◦^C, respectively. The drying and sheath gas flows were applied at 900 and 50 L/h, respectively. Automatic MS/MS fragmentation analyses of the parent ions was done via the collision-induced dissociation (CID) technique using an energy ramp from 30 to 70 eV and nitrogen gas as a collision gas. Mass spectra were acquired over the m/z range of 100–1250.

### Data processing and metabolite identification

Mass spectra were processed using MZmine 2^48^. Metabolites were tentatively identified through comparing the m/z values of the obtained adducts, retention time (Rt), and fragmentation pattern with our in-house database for *T. alexandrinum* metabolites, literature data, online databases, such as the Human Metabolome Database (HMDB)^49^, dictionary of natural products database (CRC) https://dnp.chemnetbase.com/chemical/ChemicalSearch.xhtml?dswid=9006, and others. In addition, Competitive Fragmentation Modeling-ID software (CFM-ID 4.0) was utilized for compound identification through matching the obtained experimental MS/MS fragments with those present in predicted spectral libraries^50^. Hierarchical clustering (HCA)- heat map analysis was performed using Metaboanalyst 6.0 (https://www.metaboanalyst.ca/).

### Network pharmacology analysis

Pubchem database (https://pubchem.ncbi.nlm.nih.gov/) was used to obtain the SMILES strings of annotated compounds, which could then be used as inputs to easily investigate the targets in genomic databases. Next, the STITCH (http://stitch.embl.de/, ver. 5.0), PharmMapper (https://www.lilab-ecust.cn/pharmmapper/), and Similarity Ensemble Approach (SEA) (https://sea.bkslab.org/search) databases were searched for potential molecular genes closely linked to the identified phytoconstituents with “*Homo sapiens*” species selected.

Furthermore, using the keyword “Inflammation” the genes associated with inflammations were extracted from the therapeutic target database (TTD) (http://db.idrblab.net/ttd/) and GeneCards (https://www.genecards.org/) databases. All anticipated genes implicated in human diseases and those affecting other animals are covered in detail and are easily navigable by using these integrative expert-curated databases. Next, genes shared by compound-disease combinations were identified as possible targets for *T. alexandrinum* in the management of inflammations using a Venn diagram generated by the free bioinformatics tool; Venny 2.1 (https://bioinfogp.cnb.csic.es/tools/venny/). Additionally, to construct a protein–protein interactions network (PPIs), the regulatory common targets were submitted to the String database (version 12.0) (http://string-db.org/). This open-access biological resource offers a fully referenced and integrative annotation of targeted human genes and their corresponding pathways. The major targets were extracted using this database, with the species set to *“Homo sapiens”*, and protein interactions with interaction scores >0.6 were selected, leaving all other parameters unchanged.

Ultimately, Cytoscape 3.10.2 software was used to build and analyze the pharmacological networks in order to methodically identify the critical genes and signaling pathways that *T. alexandrinum* targets in the therapy of inflammations. To determine each established network’s node’s level of contribution and importance, the Network Analyzer plug-in was used.

### Isolation of some active metabolites from *T. alexandrinum* aerial parts

The butanol fraction (BuOH) of *T. alexandrinum* extract was chosen for the isolation of active compounds based on findings from network pharmacology analysis. Butanol fraction (9 g) was chromatographed using vacuum liquid chromatography (VLC) (9 g, 6 cm × 20 cm) packed with C18 (C18-Reversed phase silica gel, Sigma-Aldrich, USA). Elution was performed starting with 100% water with a gradient increase (25%) of methanol, where five subfractions were obtained (A–E). Subfraction B (900 mg), eluted at 25% MeOH in H2O, was further purified using another C18-packed VLC (900 mg, 6 cm × 10 cm), with a methanol gradient increase of 10% in water, and similar fractions were pooled (B1–B7). The fraction (B1) eluted at 100% water (750 mg) was further separated using Sephadex (Sephadex® G-100, Sigma-Aldrich, USA) packed in a glass column eluted with methanol, yielding ten fractions, from which compound 1 (17 mg) and compound 2 (10 mg) were isolated. These two compounds were used at different concentrations for in vitro testing.

### Cell viability assay

WI38 human fibroblast cells (CCL-75) were brought from the American Type Culture Collection (ATCC, USA). MTT (3-[4,5-dimethylthiazol-2-yl]-2,5-diphenyltetrazolium bromide), dimethyl sulfoxide (DMSO), and fetal bovine serum (FBS) were obtained from GIBCO (Grand Island, NY, USA). WI38 cell suspension was seeded at a density of 3000 cells per well in 96-well plate in RPMI medium containing (10% fetal bovine and 2% l-glutamine) devoid of additional fetal bovine serum or the standard anti-inflammatory drug used in this work (piroxicam). The plates were then incubated for 72 h in a CO_2_ incubator maintained at 37 °C, 5% CO_2_, and 90% relative humidity.

Following the incubation period, the cells’ viability was measured using the MTT assay to determine the cytotoxicity of the examined samples (extract and fractions) toward WI38 cells. The MTT assay measures the reduction of the yellow tetrazolium salt MTT into the purple formazan product by viable cells^51^. Briefly, in a 96-well plate, 3000 WI38 cell/ well (ATCC: CCL-75) were plated with different concentrations of each extract/ fraction suspended in RPMI medium without fetal bovine serum or standard anti-inflammatory drugs piroxicam then the plate was incubated for 48 h in CO_2_ incubator (37°C, 5% CO_2_, and 90% relative humidity). Then, 20 µL of MTT solution was added to each well, and the plates were incubated for an additional 3 h. Subsequently, the plates were centrifuged at 1650 rpm for 10 min, and the supernatant was discarded. The formed formazan crystals were resuspended in 100 µL of DMSO, and the absorbance was measured at 570 nm using an Optima spectrophotometer to identify the safe dose that resulted in 100% cell viability.

The percentage viability was calculated using the following formula:

$$\left({\rm{AT}}{-}{\rm{Ab}}/{\rm{AC}}{-}{\rm{Ab}}\right)\times 100$$

where:

AT = mean absorbance of cells treated with different concentrations of each plant extract.

AC = mean absorbance of control, untreated cells with culture medium only.

Ab = mean absorbance of cells treated with the vehicle of the plant extract (RPMI without fetal bovine serum).

The cytotoxicity of the compound was expressed as IC50 and was calculated using GraphPad Prism software based on the percentage viability derived from serial dilutions of each plant extract.

### Real time PCR for TNF-α, IL-1β, IL-6, and INF-γ gene expression in WI38 human fibroblast cells

RNA isolation and real-time PCR assays were performed to determine gene expression levels of the inflammatory cytokines TNF-α, IL-1β, IL-6, and INF-γ as described in a previous study^52^. In brief, WI38 human fibroblast cells (5 × 10^4^) were seeded in a 12-well plate, followed by treatment with lipopolysaccharide (LPS) (GIBCO, Grand Island, NY, USA) at a concentration of 2 µg/ml. The plate was subsequently incubated for 24 h in a CO_2_ incubator maintained at 37 °C with 5% CO_2_ and 90% relative humidity. After incubation, the plate was centrifuged at 1650 rpm for 5 min, and the supernatants were discarded. Subsequently, 750 µl of the plant extract or fractions at a concentration of 100 μg/mL was added to each well. The isolated compounds and piroxicam were tested at concentrations equivalent to 1/10th of their IC50 values, identified in a cell viability assay^51^. The plate was incubated for 48 h under controlled conditions (37 °C, 5% CO_2_, 90% relative humidity). Following the incubation period, the plate was centrifuged, and the cells were subjected to RNA isolation.

RNA isolation was performed using a commercially available kit (iNtRON Biotechnology, Korea) following the manufacturer’s instructions. The extracted RNA was reverse transcribed into complementary DNA cDNA using a SensiFAST cDNA synthesis kit (Bioline, London). Quantitative polymerase chain reaction (qPCR) was then employed to amplify target genes, with glyceraldehyde-3-phosphate dehydrogenase (GAPDH) serving as a housekeeping gene.

PCR tubes were prepared by combining 12.5 µl of SensiFAST SYBR (Bioline, London), 1 µl of cDNA, 0.5 µl of 10 pmoles/ml forward primer, and 0.5 µl of 10 pmoles/ml reverse primer for each gene of interest. Nuclease-free distilled water was added to bring the final reaction volume to 20 µl.

The following primer sequences were used;

**Table:** 

Gene	Primer
TNF alpha	F-CTCTTCTGCCTGCTGCACTTTG
	R- ATGGGCTACAGGCTTGTCACTC
IL-6	F, 5′-TGAACTCCTTCTCCACAAGCG-3′
	R, 5′-TCTGAAGAGGTGAGTGGCTGTC-3′
IL1B	F, CCACAGACCTTCCAGGAGAATG
	R, GTGCAGTTCAGTGATCGTACAGG
INF-gamma	F, GAGTGTGGAGACCATCAAGGAAG
	R, TGCTTTGCGTTGGACATTCAAGTC
GAPDH	F, GGATTTGGTCGTATTGGG
	R, GGAAGATGGTGATGGGATT

The samples were loaded into the PCR system (CFX96™ Real-Time System, BIO-RAD, USA) and subjected to the following amplification program: 1 cycle of 95 °C for 10 min (initial denaturation), followed by 40 cycles of 95 °C for 15 s (denaturation), 60 °C for 30 s (annealing), and 72 °C for 30 s (extension). The cycle threshold (Ct) values of the target gene were normalized to the Ct values of the housekeeping gene (GAPDH) using the 2^−ΔΔCt^ method^53^ to calculate the fold change in gene expression, as described by the following formulas.

Expression fold levels of the gene were calculated as follows:

$$\Delta {{\rm{Ct}}}_{{\rm{normal}}}={{\rm{Ct}}}_{{\rm{normal}}{\rm{untreated}}\; {\rm{cells}}}-{{\rm{Ct}}}_{{\rm{references}}}$$

$$\Delta {{\rm{Ct}}}_{{\rm{tested}}\; {\rm{plant}}\; {\rm{extract}}}={{\rm{Ct}}}_{{\rm{tested}}\; {\rm{plant}}\; {\rm{extract}}-{\rm{treated}}\; {\rm{cells}}}-{{\rm{Ct}}}_{{\rm{references}}}$$

$$\Delta {{\rm{Ct}}}_{{\rm{induced}}}\,=-\,{{\rm{Ct}}}_{{\rm{LPS}}-{\rm{exposed}}\; {\rm{cells}}}-{{\rm{Ct}}}_{{\rm{references}}}$$

In case of genes:

$$\Delta \Delta {{\rm{Ct}}}_{{\rm{tested}}\; {\rm{plant}}\; {\rm{extract}}}=\Delta {{\rm{Ct}}}_{{\rm{tested}}\; {\rm{plant}}\; {\rm{extract}}}{{\mbox{-}\; \Delta }}{{\rm{Ct}}}_{{\rm{normal}}}$$

$$\Delta \Delta {{\rm{Ct}}}_{{\rm{induced}}}=\Delta {{\rm{Ct}}}_{{\rm{induced}}}{{\mbox{-}\; \Delta }}{{\rm{Ct}}}_{{\rm{normal}}}$$

In case of GAPDH:

$$\Delta \Delta {{\rm{Ct}}}_{{\rm{tested}}\; {\rm{plant}}\; {\rm{extract}}}=\Delta {{\rm{Ct}}}_{{\rm{normal}}}\,-{{\Delta }}{{\rm{Ct}}}_{{\rm{tested}}\; {\rm{plant}}\; {\rm{extract}}}$$

$$\Delta \Delta {{\rm{Ct}}}_{{\rm{induced}}}=\Delta {{\rm{Ct}}}_{{\rm{normal}}}-{{\Delta }}{{\rm{Ct}}}_{{\rm{induced}}}$$

$${\rm{Fold}}\; {\rm{change}}\; {\rm{in}}\; {\rm{gene}}\; {\rm{expression}}=\log ({2}^{-\Delta \Delta {\rm{CT}}})$$

Where: Ct _tested plant extract_: threshold cycle value of genes of extracted mRNA of plant extract treated-LPS-stimulated WI38 human fibroblast cells, which is defined as the cycle number at which the fluorescence generated within a reaction crosses the fluorescence threshold.

Ct _reference_: threshold cycle value of GAPDH, which is used for normalization.

Ct _normal_: threshold cycle value of genes of extracted mRNA of untreated control WI38 cells.

Ct _induced_: threshold cycle value of the gene of extracted mRNA of LPS-stimulated WI38 cells.

### Statistical analysis

All results were expressed as the mean ± SD of triplicates. Experimental data were analyzed using one-way analysis of variance (ANOVA) for group comparison and post hoc test (Tukey) for pairwise comparison using IBM SPSS software version 20.0. (Armonk, NY: IBM Corp). Values of *p* < 0.05 were considered statistically significant.

## Supplementary information

Supplementary materials

Table S1

## Data Availability

No datasets were generated or analysed during the current study.
