# Supplementary material for: Network pharmacology and UPLC/MS/MS metabolic profiling unveil the anti-inflammatory potential of Trifolium alexandrinum
Source: NPJ Sci Food. 2025 Jun 14;9:102. doi: 10.1038/s41538-025-00459-y (PMC12167384; doi:10.1038/s41538-025-00459-y)
Supplement: Supplementary file 1 — Supplementary materials [file 41538_2025_459_MOESM1_ESM.docx]

**Network pharmacology and UPLC/MS/MS metabolic profiling unveil the anti-inflammatory potential of *Trifolium alexandrinum***

**Rahma SR. Mahrous ^1^, Hoda Fathy ^1^,** **Doaa A. Ghareeb ^2,3,4^, Ali S. Abdel‑Hamid^3^ and Reham S. Ibrahim ^1,^** ^*^

^1^ Department of Pharmacognosy, Faculty of Pharmacy, Alexandria university, Alexandria, Egypt

^2^ Bio-Screening and Preclinical Trials Lab, Biochemistry Department, Faculty of Science, Alexandra University, Alexandria, Egypt.

^3^ Center of Excellence for Drug Preclinical Studies (CE-DPS), Pharmaceutical and Fermentation Industry Development Center, City of Scientific Research & Technological Applications, New Borg El Arab, Alexandria, Egypt

^4^ Research Projects unit, Pharos University in Alexandria; Canal El Mahmoudia Street, Beside Green Plaza Complex 21648, Alexandria, Egypt.

**Supplementary material**

**Results**

| **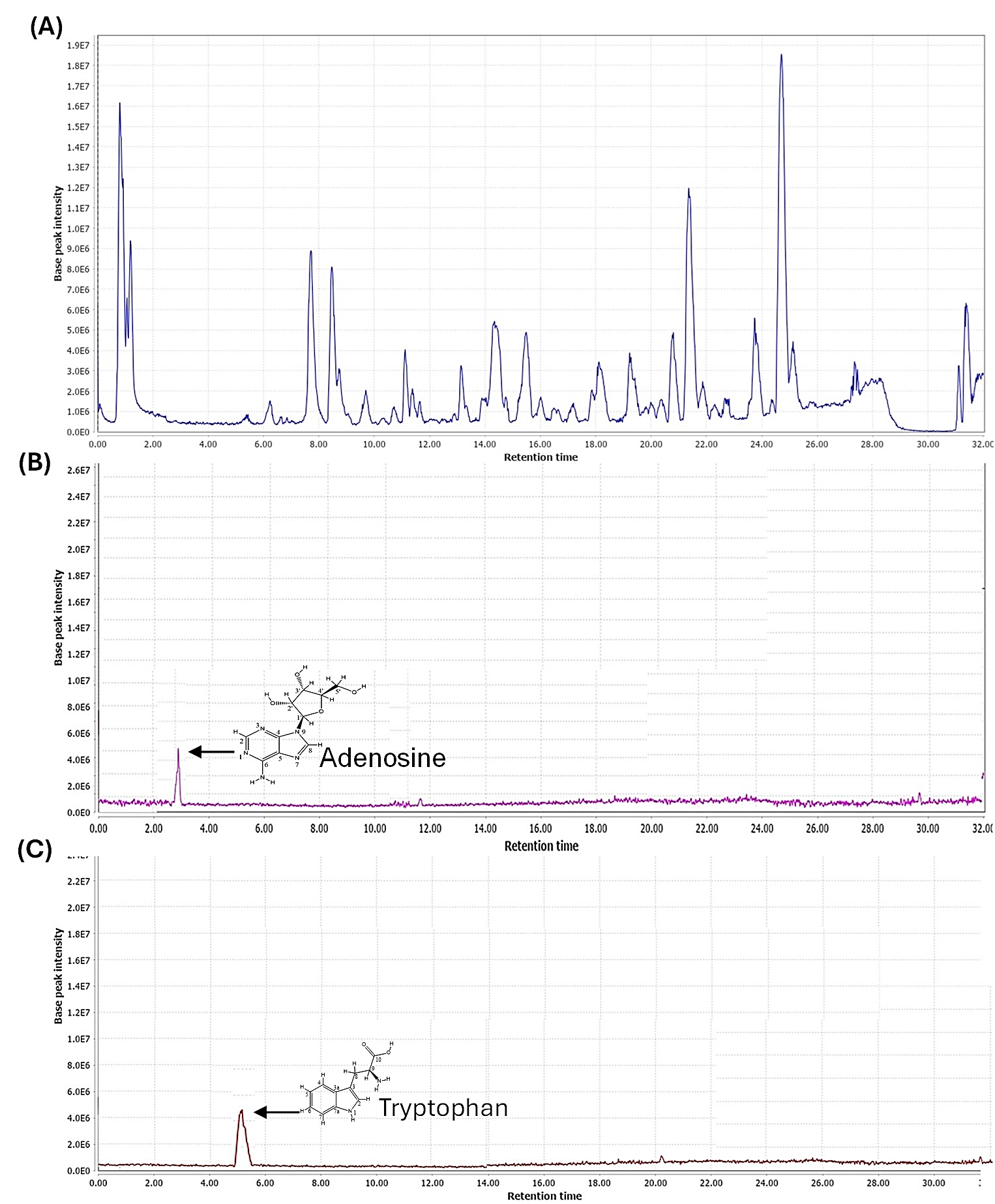** |
| --- |
| **Fig S1. UPLC-MS base peak chromatogram of (A) *T. alexandrinum* extract and the isolated compounds (B) adenosine, and (C) tryptophan in positive ion mode under the same conditions referred to in the methods section.** |

**Table S1. Potential protein targets of *T. alexandrinum*: please refer to the excel file.**

**Table S2. *T. alexandrinum* four solvent fractions network node topological parameters**

| **Compound name** | **Betweenness Centrality** | **Closeness Centrality** | **Degree** |
| --- | --- | --- | --- |
| Hexane | 0.385001 | 0.468876 | 287 |
| Dichloromethane | 0.096664 | 0.399145 | 158 |
| Ethylacetate | 0.052633 | 0.380293 | 102 |
| Butanol | 0.77476059 | 0.63623978 | 408 |

**Table S3. KEGG pathway analysis of potential target gene functions**

| **#term ID** | **term description** | **observed gene count** | **false discovery rate (p value)** | **matching proteins in your network (labels)** |
| --- | --- | --- | --- | --- |
| hsa00590 | Arachidonic acid metabolism | 15 | 2.58E-10 | CYP4F2,LTA4H,ALOX12,CBR1,ALOX15,HPGDS,PTGS1,PLA2G4A,CYP2C19,ALOX5,ALOX15B,PLA2G2A,PLA2G10,EPHX2,PLA2G4C |
| hsa05418 | Fluid shear stress and atherosclerosis | 19 | 8.32E-10 | TNFRSF1A,MMP2,PLAT,NFKB1,NOS3,FOS,NQO1,HSP90AA1,GSTA1,CTSL,MAP3K7,JUN,MMP9,NFE2L2,GSTP1,RELA,MAP2K6,IKBKG,AKT3 |
| hsa05161 | Hepatitis B | 20 | 2.53E-09 | NFKB1,TLR2,EP300,PRKCG,FOS,CXCL8,CASP3,SMAD3,MAP3K7,JUN,MMP9,PCNA,RELA,IL6,CREB1,PRKCA,MAP2K6,IKBKG,PRKCB,AKT3 |
| hsa05206 | MicroRNAs in cancer | 20 | 2.53E-09 | SIRT1,NFKB1,EP300,PRKCG,MMP16,PRKCE,CASP3,GLS,CDC25C,MDM4,MCL1,MMP9,PLAU,HDAC1,ABCC1,PRKCA,HDAC2,ABCB1,ESR1,PRKCB |
| hsa04724 | Glutamatergic synapse | 17 | 4.57E-09 | SLC1A1,PRKCG,SLC1A3,SLC1A2,PRKACA,GLS,GRIK1,GNG2,GRM3,PLA2G4A,GRIK3,GNB1,GRIK2,PRKCA,GRIA1,PLA2G4C,PRKCB |
| hsa04210 | Apoptosis | 18 | 5.19E-09 | TNFRSF1A,CTSH,NFKB1,CTSC,CTSK,CAPN2,FOS,CASP3,CTSL,CTSB,CTSS,MCL1,JUN,RELA,PTPN13,CAPN1,IKBKG,AKT3 |
| hsa04066 | HIF-1 signaling pathway | 15 | 6.97E-08 | NFKB1,EP300,PRKCG,NOS3,NOS2,EGLN1,NPPA,PDK1,RELA,IL6,PRKCA,SLC2A1,HIF1A,PRKCB,AKT3 |
| hsa04931 | Insulin resistance | 15 | 0.000000104 | TNFRSF1A,NFKB1,NOS3,PRKCE,SLC2A4,SLC2A2,PRKCD,PTPN1,RELA,PPARA,IL6,CREB1,SLC2A1,PRKCB,AKT3 |
| hsa00140 | Steroid hormone biosynthesis | 12 | 0.000000114 | STS,HSD11B2,CYP1A2,COMT,CYP17A1,AKR1C4,AKR1C2,AKR1C1,IL1B,CYP19A1,ESR1,CYP3A4 |
| hsa04926 | Relaxin signaling pathway | 16 | 0.000000114 | MMP2,NFKB1,NOS3,FOS,PRKACA,MMP1,NOS2,GNG2,JUN,MMP9,EDNRB,GNB1,RELA,CREB1,PRKCA,AKT3 |
| hsa04750 | Inflammatory mediator regulation of TRP channels | 14 | 0.000000123 | PTGER2,ALOX12,PRKCG,PTGER4,PRKCE,PRKACA,PRKCH,PRKCD,PLA2G4A,PRKCA,TRPV1,MAP2K6,PLA2G4C,PRKCB |
| hsa04668 | TNF signaling pathway | 15 | 0.000000142 | TNFRSF1A,NFKB1,MMP3,FOS,CASP3,RPS6KA4,MAP3K7,JUN,MMP9,RELA,IL6,CREB1,MAP2K6,IKBKG,AKT3 |
| hsa04024 | cAMP signaling pathway | 19 | 0.000000422 | NFKB1,GHSR,PTGER2,EP300,FOS,PRKACA,EDNRA,PTGER3,SUCNR1,RYR2,JUN,NPPA,GABBR1,HCAR2,RELA,PPARA,CREB1,GRIA1,AKT3 |
| hsa01523 | Antifolate resistance | 9 | 0.000000426 | NFKB1,ATIC,ALOX12,ABCC1,RELA,IL6,IKBKG,ABCC2,ABCG2 |
| hsa04657 | IL-17 signaling pathway | 13 | 0.000000596 | NFKB1,MMP3,FOS,CXCL8,CASP3,MMP1,HSP90AA1,MAP3K7,JUN,MMP9,RELA,IL6,IKBKG |
| hsa04614 | Renin-angiotensin system | 8 | 0.000000706 | CTSG,CMA1,ACE,CPA3,ANPEP,KLK1,ACE2,MME |
| hsa00790 | Folate biosynthesis | 8 | 0.00000108 | TPH1,AKR1B1,CBR1,ALPG,ALPI,AKR1B10,ALPL,TH |
| hsa04071 | Sphingolipid signaling pathway | 14 | 0.00000108 | TNFRSF1A,NFKB1,OPRD1,ADORA3,PRKCG,NOS3,PRKCE,SPHK1,ABCC1,RELA,PRKCA,SPHK2,PRKCB,AKT3 |
| hsa04972 | Pancreatic secretion | 13 | 0.00000108 | CPA1,CPB2,PRKCG,CA2,KCNMA1,CPA3,CTRB1,RYR2,PLA2G2A,PLA2G10,PRKCA,AMY2A,PRKCB |
| hsa05030 | Cocaine addiction | 10 | 0.00000108 | NFKB1,PRKACA,MAOA,GRM3,JUN,MAOB,TH,RELA,CREB1,PDYN |
| hsa05170 | Human immunodeficiency virus 1 infection | 18 | 0.00000112 | TNFRSF1A,NFKB1,TLR2,PRKCG,FOS,CASP3,CDC25C,GNG2,MAP3K7,JUN,GNB1,CDK1,RELA,PRKCA,MAP2K6,IKBKG,PRKCB,AKT3 |
| hsa04620 | Toll-like receptor signaling pathway | 13 | 0.00000125 | NFKB1,TLR2,CTSK,FOS,CXCL8,TLR9,MAP3K7,JUN,RELA,IL6,MAP2K6,IKBKG,AKT3 |
| hsa04974 | Protein digestion and absorption | 13 | 0.00000125 | CPA1,CPB2,SLC1A1,CPA3,COL18A1,DPP4,CTRB1,XPNPEP2,SLC15A1,ACE2,MME,SLC1A5,KCNN4 |
| hsa00980 | Metabolism of xenobiotics by cytochrome P450 | 11 | 0.00000173 | CBR1,HPGDS,GSTA1,CYP1A2,AKR1C1,IL1B,GSTP1,CYP1B1,EPHX1,CYP2D6,CYP3A4 |
| hsa00591 | Linoleic acid metabolism | 8 | 0.00000207 | ALOX15,CYP1A2,PLA2G4A,CYP2C19,PLA2G2A,PLA2G10,PLA2G4C,CYP3A4 |
| hsa04020 | Calcium signaling pathway | 17 | 0.00000207 | PRKCG,NOS3,TACR3,PRKACA,SPHK1,EDNRA,NOS2,PTGER3,P2RX4,RYR2,PTGFR,CYSLTR1,EDNRB,TBXA2R,PRKCA,SPHK2,PRKCB |
| hsa04728 | Dopaminergic synapse | 14 | 0.00000207 | PRKCG,FOS,PRKACA,GNG2,MAOA,COMT,MAOB,GNB1,TH,CREB1,PRKCA,GRIA1,PRKCB,AKT3 |
| hsa04976 | Bile secretion | 12 | 0.00000207 | NR0B2,CA2,HMGCR,PRKACA,SLC22A8,SLC2A1,NR1H4,ABCB1,EPHX1,ABCC2,ABCG2,CYP3A4 |
| hsa04270 | Vascular smooth muscle contraction | 14 | 0.0000032 | PRKCG,KCNMA1,PRKCE,PRKACA,EDNRA,PRKCH,PRKCD,PLA2G4A,NPPA,PLA2G2A,PLA2G10,PRKCA,PLA2G4C,PRKCB |
| hsa04915 | Estrogen signaling pathway | 14 | 0.0000034 | MMP2,NOS3,FOS,PRKACA,PRKCD,HSP90AA1,TNF,JUN,MMP9,GABBR1,CREB1,OPRM1,IFNG,AKT3 |
| hsa05204 | Chemical carcinogenesis | 11 | 0.00000343 | CBR1,HPGDS,GSTA1,CYP1A2,CYP2C19,AKR1C2,IL1B,GSTP1,ESR1,EPHX1,CYP3A4 |
| hsa05215 | Prostate cancer | 12 | 0.00000462 | PLAT,NFKB1,EP300,MMP3,HSP90AA1,MMP9,PLAU,GSTP1,RELA,CREB1,IKBKG,AKT3 |
| hsa04010 | MAPK signaling pathway | 20 | 0.00000542 | TNFRSF1A,NFKB1,PRKCG,FOS,PRKACA,CASP3,RPS6KA4,MAPT,PLA2G4A,MAP3K7,JUN,RELA,PRKCA,NR4A1,PGF,MAP2K6,PLA2G4C,IKBKG,PRKCB,AKT3 |
| hsa04380 | Osteoclast differentiation | 13 | 0.00000596 | TNFRSF1A,NFKB1,CTSK,PPARG,FOS,MAP3K7,JUN,RELA,CREB1,MITF,MAP2K6,IKBKG,AKT3 |
| hsa00350 | Tyrosine metabolism | 8 | 0.00000598 | MIF,TYR,AOC3,MAOA,COMT,MAOB,TH,DBH |
| hsa04911 | Insulin secretion | 11 | 0.00000606 | FFAR1,PRKCG,KCNMA1,PRKACA,SLC2A2,RYR2,CREB1,PRKCA,SLC2A1,PRKCB,KCNN4 |
| hsa05146 | Amoebiasis | 12 | 0.0000061 | CTSG,NFKB1,TLR2,PRKCG,CXCL8,PRKACA,CASP3,NOS2,RELA,IL6,PRKCA,PRKCB |
| hsa05152 | Tuberculosis | 15 | 0.00000614 | TNFRSF1A,NFKB1,TLR2,EP300,CASP3,SPHK1,NOS2,HSPD1,TLR9,CTSS,RELA,IL6,CREB1,SPHK2,AKT3 |
| hsa04151 | PI3K-Akt signaling pathway | 22 | 0.00000683 | NFKB1,IL2,TLR2,NOS3,YWHAG,GNG2,HSP90AA1,MCL1,LPAR3,LPAR1,GNB1,RELA,IL6,CREB1,PRKCA,PIK3CG,NR4A1,LPAR2,PGF,PHLPP2,IKBKG,AKT3 |
| hsa05145 | Toxoplasmosis | 12 | 0.00000693 | TNFRSF1A,NFKB1,TLR2,CASP3,NOS2,MAP3K7,ALOX5,RELA,PIK3CG,MAP2K6,IKBKG,AKT3 |
| hsa04072 | Phospholipase D signaling pathway | 14 | 0.00000805 | CXCL8,F2,SPHK1,GRM3,PLA2G4A,LPAR3,PTGFR,LPAR1,PRKCA,PIK3CG,LPAR2,SPHK2,PLA2G4C,AKT3 |
| hsa04666 | Fc gamma R-mediated phagocytosis | 11 | 0.0000121 | PRKCG,PRKCE,SPHK1,PRKCD,PLA2G4A,PRKCA,PTPRC,SPHK2,PLA2G4C,PRKCB,AKT3 |
| hsa00380 | Tryptophan metabolism | 8 | 0.0000135 | TPH1,KYNU,MAOA,CYP1A2,MAOB,IL1B,TDO2,ESR1 |
| hsa04370 | VEGF signaling pathway | 9 | 0.0000135 | PRKCG,NOS3,SPHK1,PLA2G4A,PRKCA,SPHK2,PLA2G4C,PRKCB,AKT3 |
| hsa05130 | Pathogenic Escherichia coli infection | 15 | 0.0000215 | TNFRSF1A,NFKB1,FOS,CXCL8,F2,CASP3,NLRP3,MAP3K7,JUN,LPAR1,RELA,IL6,PTPN6,LPAR2,IKBKG |
| hsa05010 | Alzheimer disease | 21 | 0.0000246 | TNFRSF1A,HSD17B10,NFKB1,PSMB1,NOX4,APP,CAPN2,CASP3,BACE1,NOS2,MAPT,MT-ND4,PSMB5,AGER,SDHB,RELA,IL6,MME,CAPN1,AKT3,SNCA |
| hsa05205 | Proteoglycans in cancer | 15 | 0.0000315 | MMP2,TLR2,PRKCG,PRKACA,CASP3,PLAUR,CTSL,MMP9,PLAU,PTPN6,IFNG,PRKCA,HIF1A,PRKCB,AKT3 |
| hsa00982 | Drug metabolism - cytochrome P450 | 9 | 0.0000325 | HPGDS,GSTA1,MAOA,CYP1A2,CYP2C19,MAOB,GSTP1,CYP2D6,CYP3A4 |
| hsa04921 | Oxytocin signaling pathway | 13 | 0.0000332 | PRKCG,NOS3,FOS,PRKACA,RYR2,PLA2G4A,JUN,NPPA,TRPM2,PRKCA,PIK3CG,PLA2G4C,PRKCB |
| hsa05202 | Transcriptional misregulation in cancer | 14 | 0.0000332 | PLAT,NFKB1,PAX8,PPARG,MMP3,CXCL8,MMP9,PLAU,HDAC1,IGFBP3,RELA,IL6,MITF,HDAC2 |
| hsa04924 | Renin secretion | 9 | 0.0000383 | PTGER2,KCNMA1,ACE,PTGER4,PRKACA,EDNRA,CTSB,NPPA,CREB1 |
| hsa04913 | Ovarian steroidogenesis | 8 | 0.0000427 | PRKACA,PLA2G4A,CYP17A1,ALOX5,IL1B,CYP19A1,PLA2G4C,ESR1 |
| hsa04917 | Prolactin signaling pathway | 9 | 0.0000462 | NFKB1,FOS,SLC2A2,TNF,CYP17A1,TH,RELA,IFNG,AKT3 |
| hsa05230 | Central carbon metabolism in cancer | 9 | 0.0000462 | SLC7A5,GLS,SLC2A2,SIRT3,PDK1,SLC2A1,HIF1A,SLC1A5,AKT3 |
| hsa04725 | Cholinergic synapse | 11 | 0.0000492 | PRKCG,FOS,PRKACA,GNG2,CHAT,GNB1,CREB1,PRKCA,PIK3CG,PRKCB,AKT3 |
| hsa05140 | Leishmaniasis | 9 | 0.0000492 | NFKB1,TLR2,FOS,NOS2,MAP3K7,JUN,RELA,PTPN6,PRKCB |
| hsa05203 | Viral carcinogenesis | 14 | 0.0000602 | NFKB1,EP300,HDAC11,YWHAG,PRKACA,CASP3,JUN,HDAC1,HDAC8,CDK1,RELA,CREB1,HDAC2,IKBKG |
| hsa05166 | Human T-cell leukemia virus 1 infection | 15 | 0.0000629 | TNFRSF1A,NFKB1,IL2,EP300,FOS,PRKACA,SMAD3,KAT5,JUN,RELA,IL6,CREB1,SLC2A1,IKBKG,AKT3 |
| hsa04935 | Growth hormone synthesis, secretion and action | 11 | 0.000084 | GHSR,EP300,PRKCG,FOS,PRKACA,IGFBP3,CREB1,PRKCA,MAP2K6,PRKCB,AKT3 |
| hsa04919 | Thyroid hormone signaling pathway | 11 | 0.0001 | EP300,PRKCG,PRKACA,HDAC1,IFNG,PRKCA,SLC2A1,HDAC2,HIF1A,PRKCB,AKT3 |
| hsa04659 | Th17 cell differentiation | 10 | 0.00011 | NFKB1,IL2,FOS,SMAD3,HSP90AA1,JUN,RELA,IL6,HIF1A,IKBKG |
| hsa04014 | Ras signaling pathway | 15 | 0.00012 | NFKB1,PRKCG,PRKACA,GNG2,PLA2G4A,GNB1,PLA2G2A,RELA,PLA2G10,PRKCA,PGF,PLA2G4C,IKBKG,PRKCB,AKT3 |
| hsa04660 | T cell receptor signaling pathway | 10 | 0.00012 | NFKB1,IL2,FOS,MAP3K7,JUN,RELA,PTPN6,PTPRC,IKBKG,AKT3 |
| hsa05034 | Alcoholism | 12 | 0.00012 | HDAC11,PRKACA,GNG2,MAOA,HDAC1,HDAC8,MAOB,GNB1,TH,CREB1,HDAC2,PDYN |
| hsa00910 | Nitrogen metabolism | 5 | 0.00015 | CA2,CA4,CA6,CA9,CA1 |
| hsa05323 | Rheumatoid arthritis | 9 | 0.00016 | TLR2,CTSK,MMP3,FOS,CXCL8,MMP1,CTSL,JUN,IL6 |

| 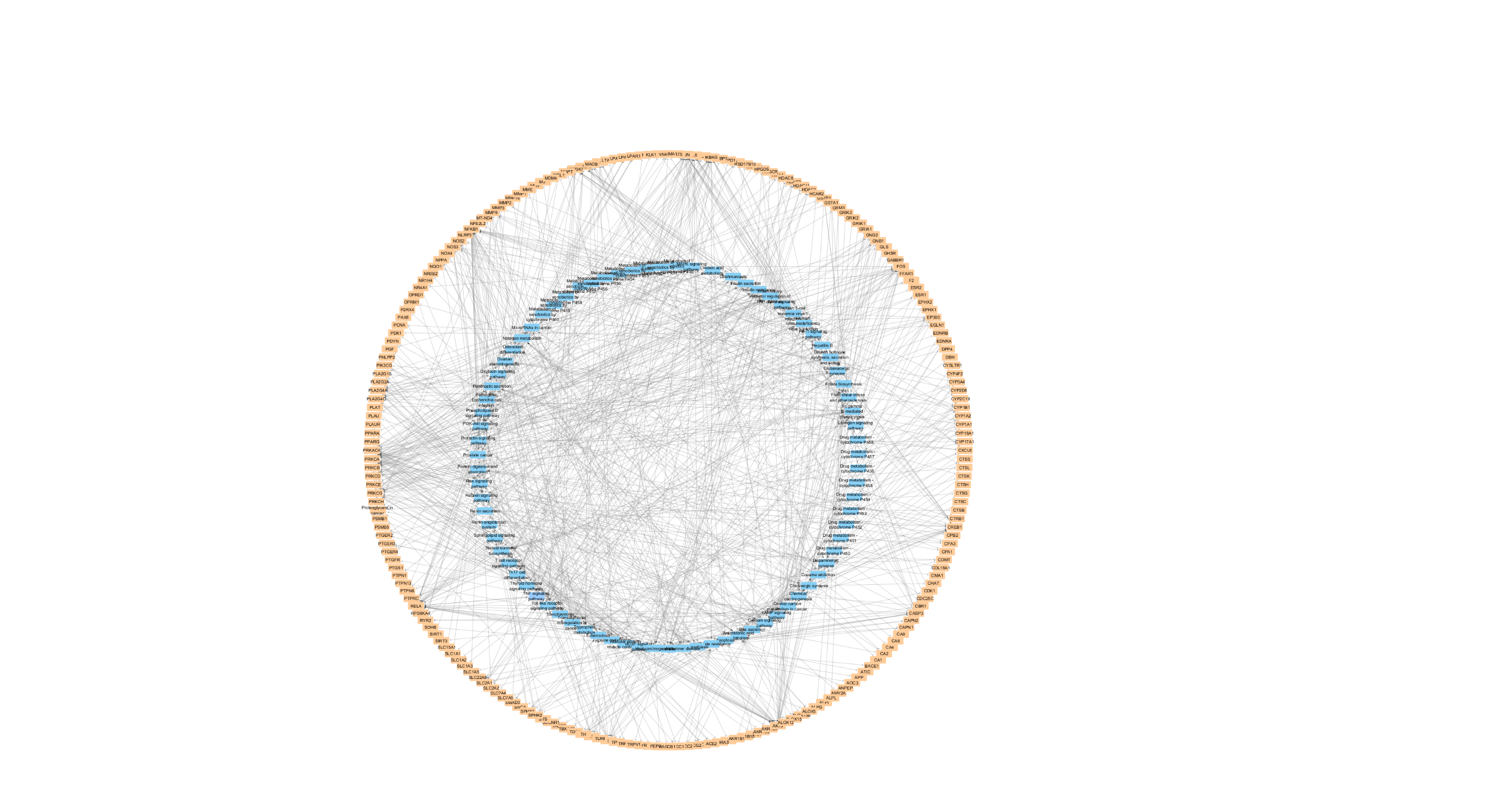 |
| --- |
| **Fig. S2 Target gene-pathway network (genes are colored in orange, pathways are colored in blue).** |

**
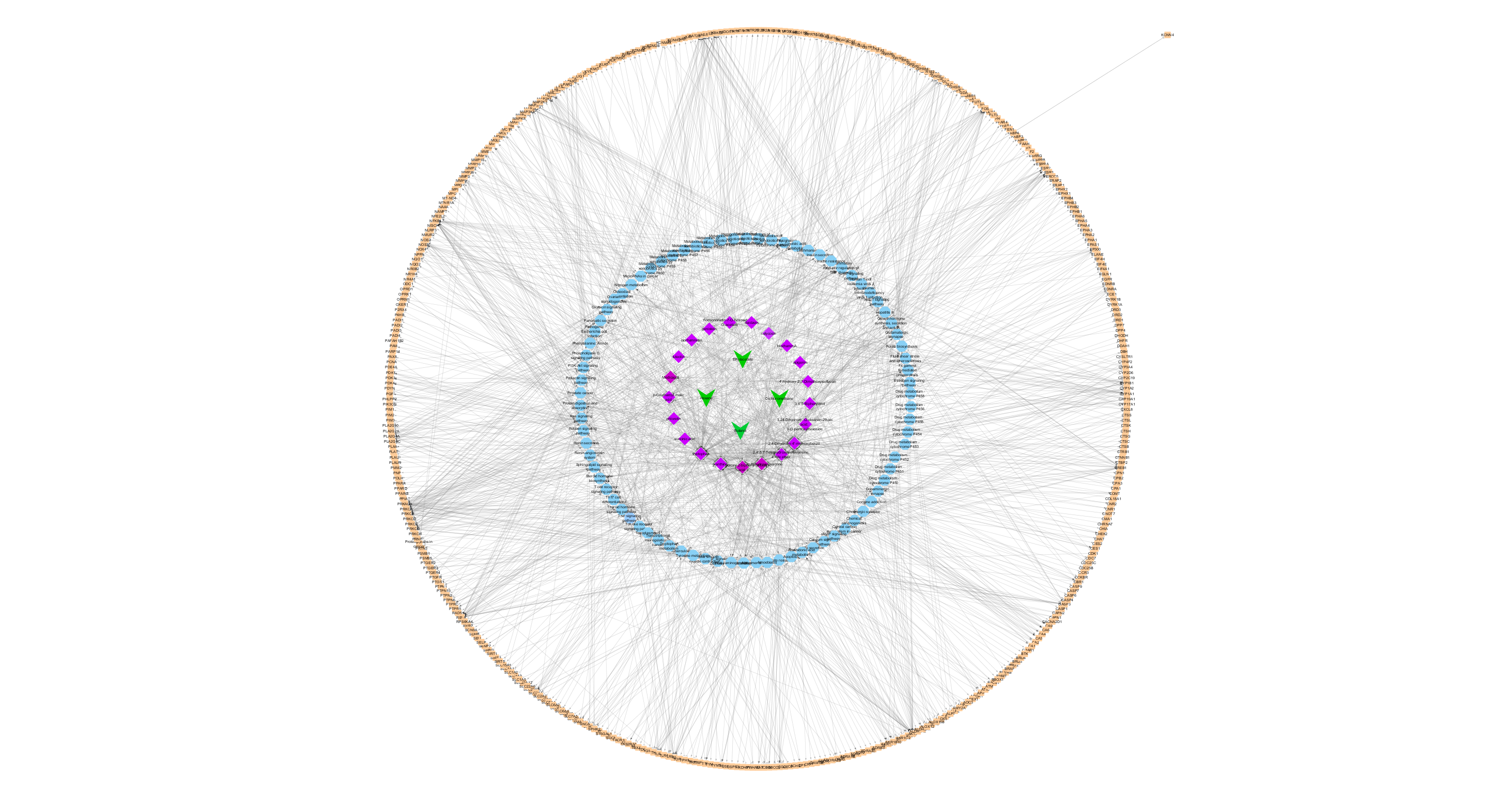
****Fig. S3 Fraction-compound–target–pathway network of *T. alexandrinum* (Fraction are colored in green, compounds in purple, targets in orange, and pathways are colored in blue).**

| 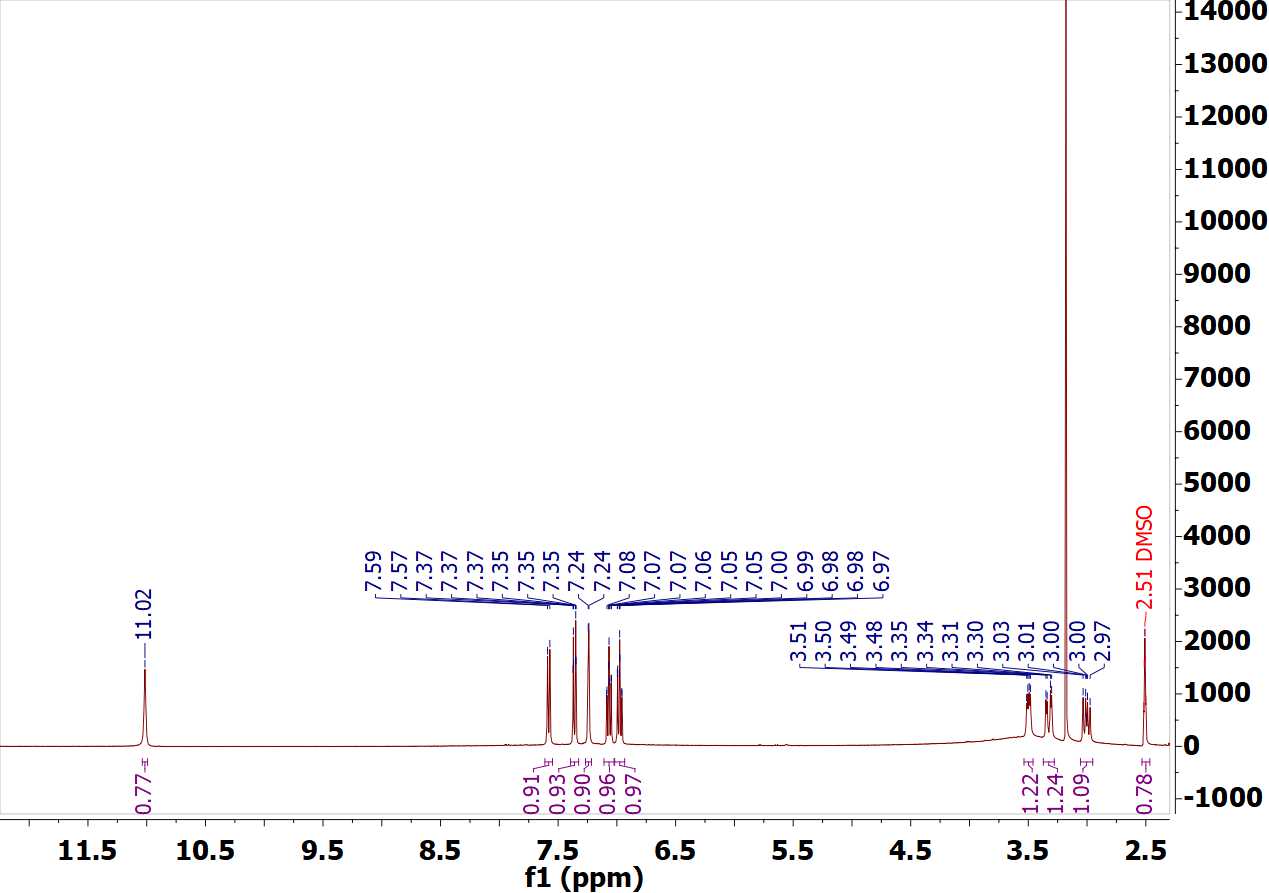 |
| --- |
| **Fig. S4 ^1^H-NMR spectrum of tryptophan (1) in DMSO, d6 at 400 MHz** |
|    |
| **Fig. S5 ^13^C-DEPTQ spectrum of tryptophan (1) in DMSO, d6 at 100 MHz** |

| **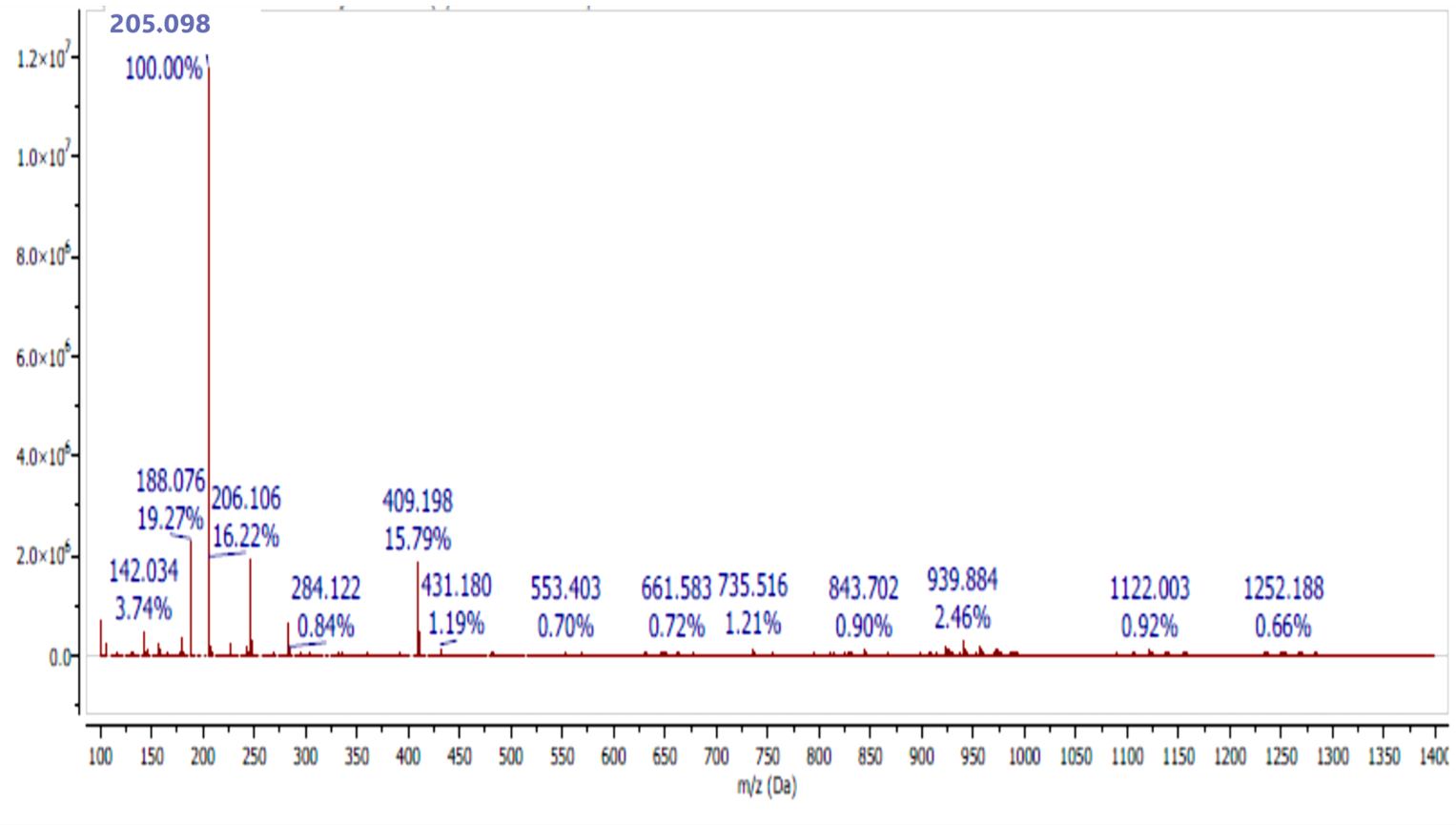** |
| --- |
| **Fig. S6 HRESI-MS spectrum of tryptophan (1) in positive ion mode** |

| 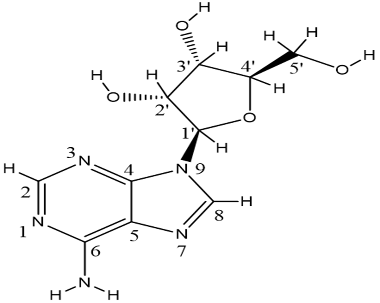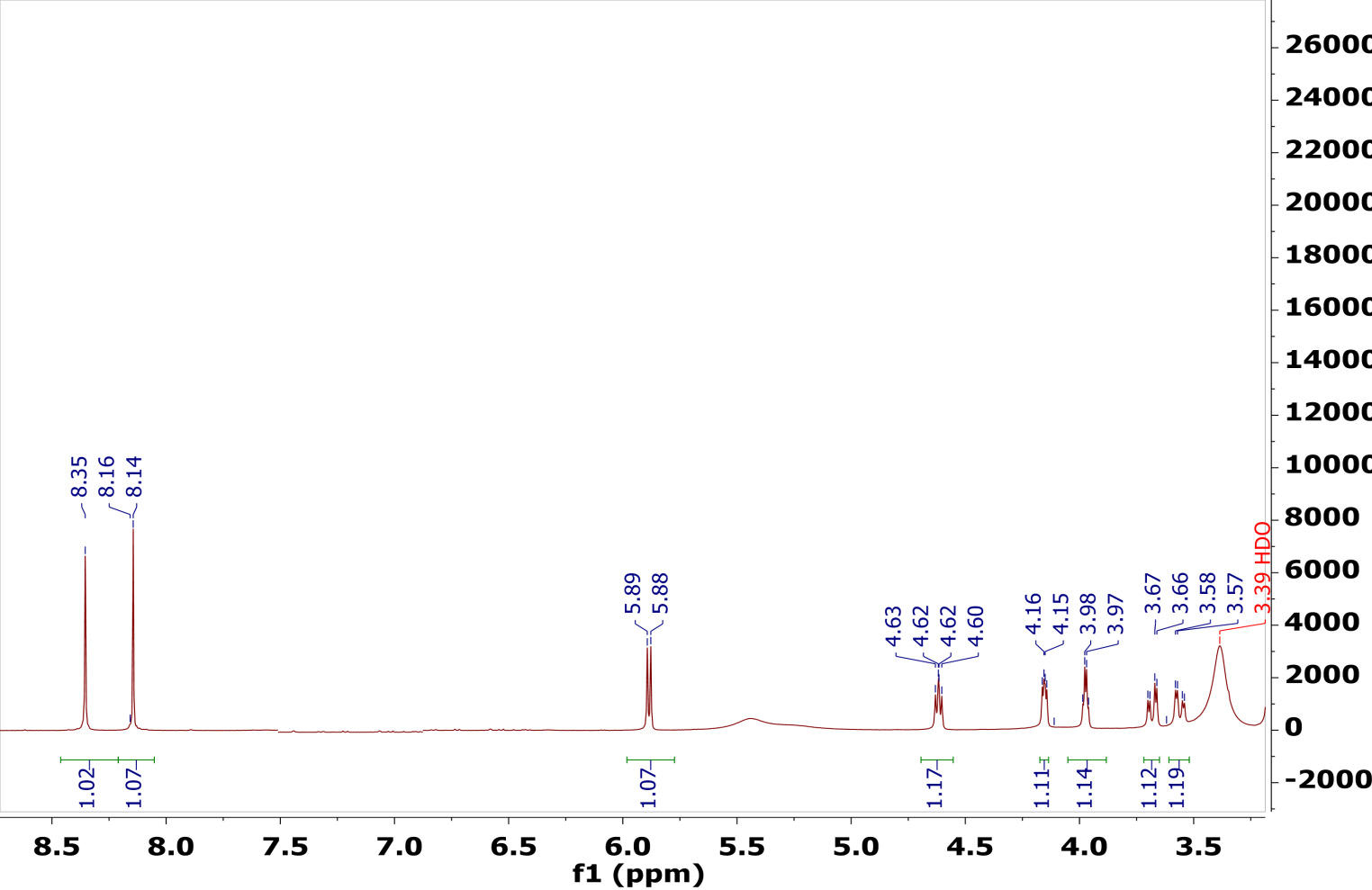 |
| --- |
| **Fig. S7 ^1^H-NMR spectrum of adenosine (2) in DMSO, d6 at 400 MHz** |
| 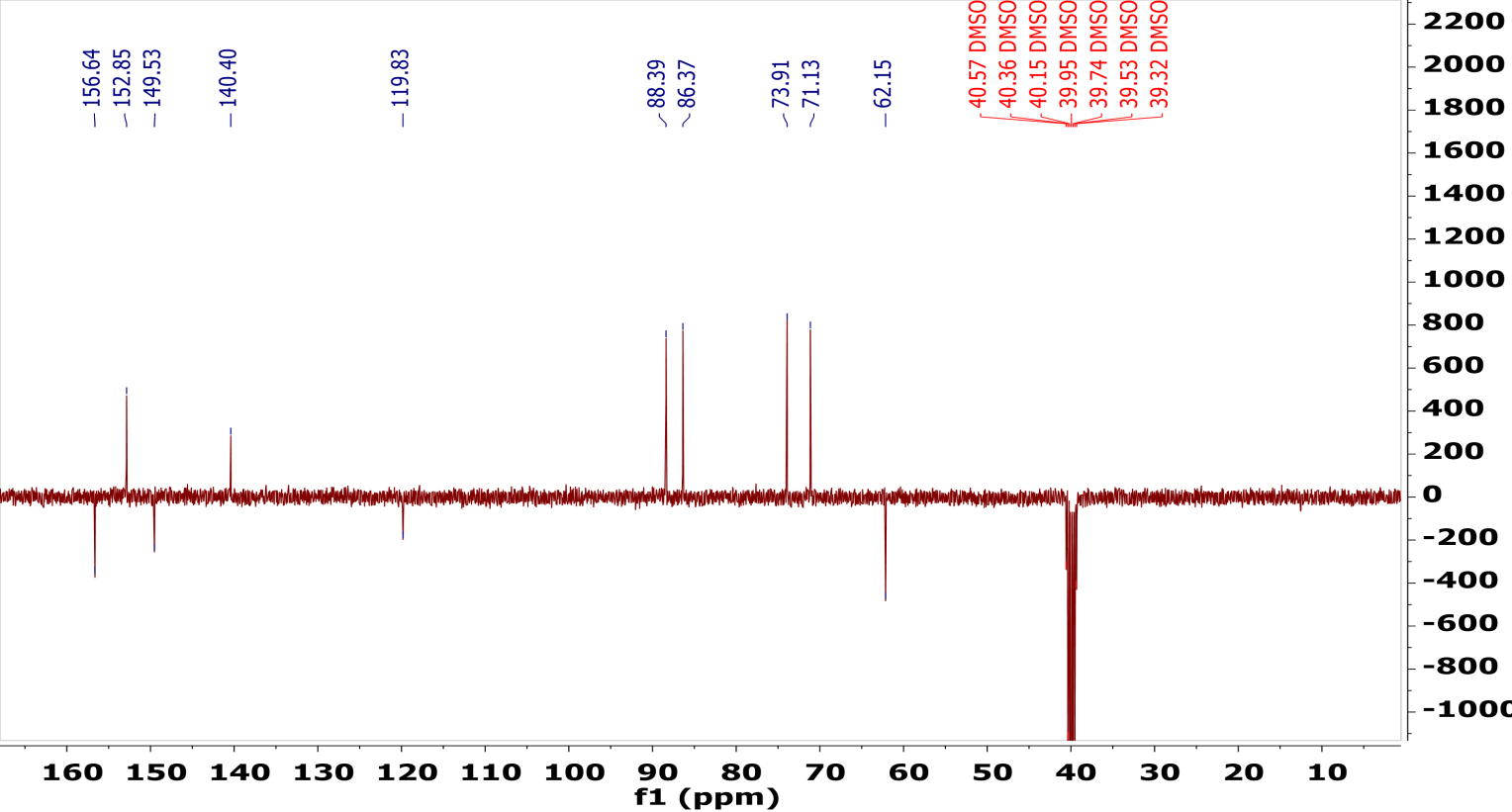  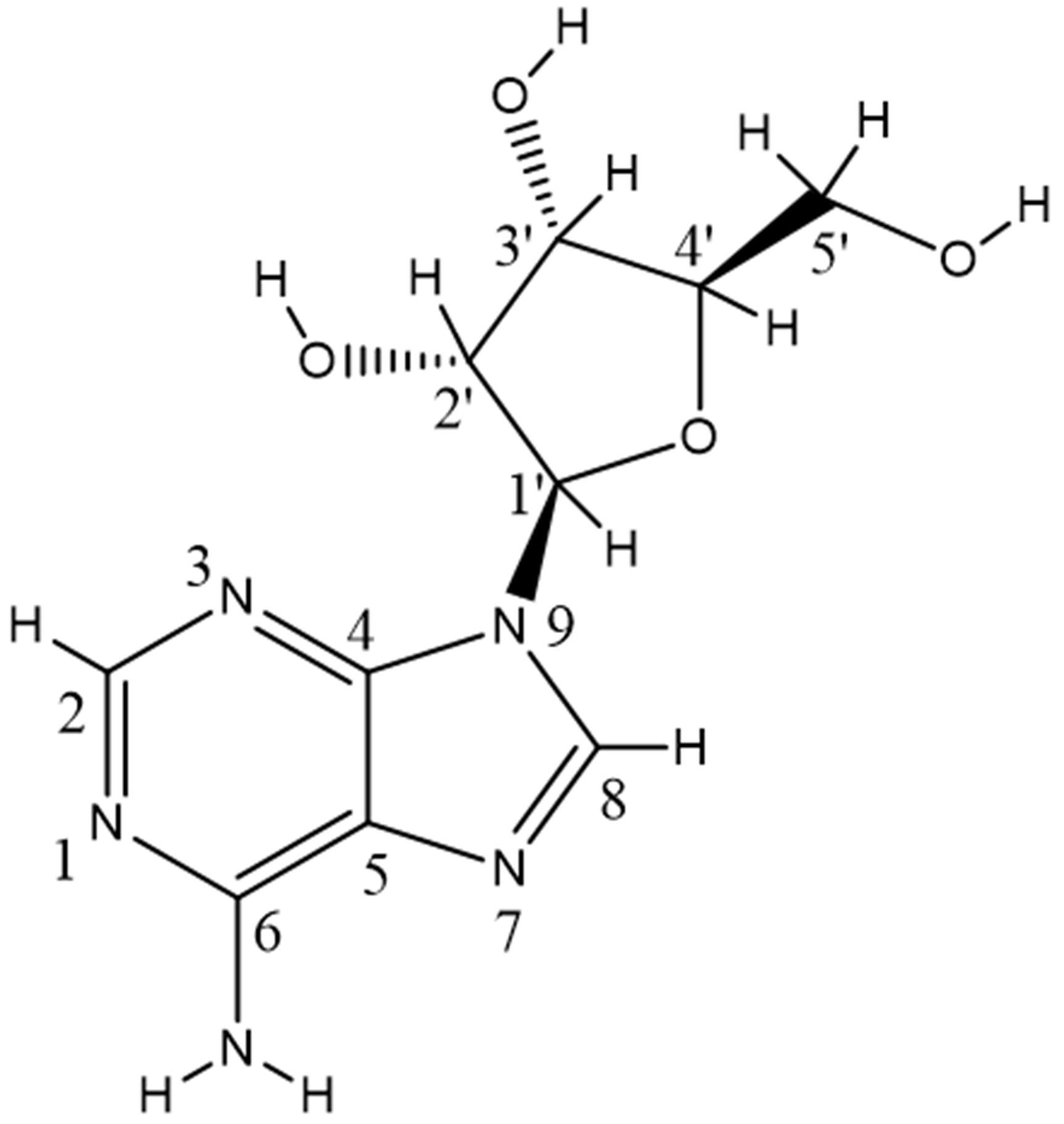 |
| **Fig. S8 ^13^C-DEPTQ spectrum of adenosine (2) in DMSO, d6 at 100 MHz** |
| **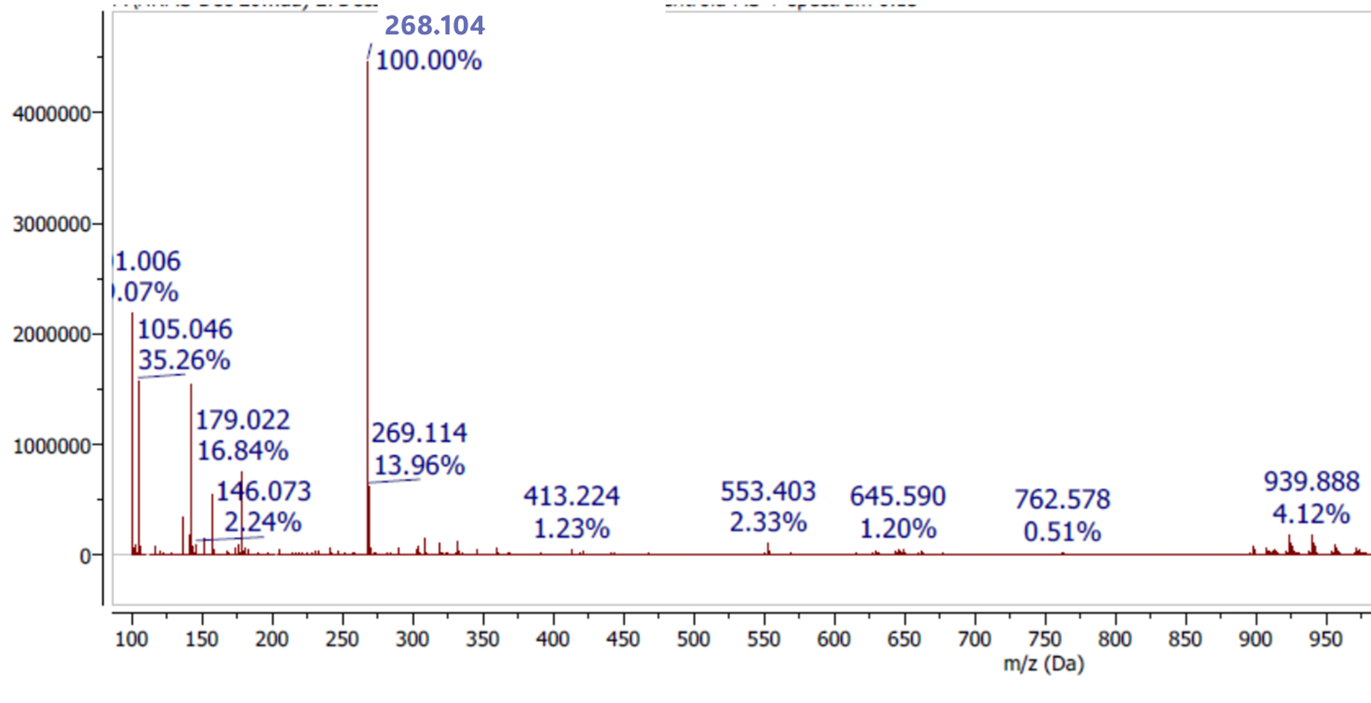** |
| **Fig. S9 HRESI-MS spectrum of adenosine (2) in positive ion mode** |

**Table S4. The effect of T. *alexandrinum* total extract, solvent fractions, and isolated compounds on TNF-α, IL-1β, IL-6, and INF- γ expression levels in LPS-induced WI38 cells ^#^**

|  | **TNF-α** | **IL-1β** | **IL-6** | **INF-γ** |
| --- | --- | --- | --- | --- |
| **Control** | 0.51 ± 0.035 | 0.65 ± 0.09 | 0.53 ± 0.089 | 0.53 ± 0.071 |
| **LPS (2 μg/ml)** | 3.07**^#^** ± 0.004 | 3.25**^#^** ± 0.28 | 4.20**^#^** ± 0.14 | 2.93**^#^** ± 0.03 |
| **Total Extract -ETOH** | 2.43* ± 0.018 | 2.13* ± 0.03 | 2.89* ± 0.07 | 1.54* ± 0.04 |
| **Hex** | 2.89* ± 0.003 | 2.61* ± 0.05 | 3.45* ± 0.01 | 2.35* ± 0.03 |
| **CH2Cl2** | 2.99* ± 0.003 | 2.87* ± 0.003 | 3.64* ± 0.003 | 2.19* ± 0.004 |
| **ETOAc** | 2.65* ± 0.037 | 2.65* ± 0.001 | 3.26* ± 0.004 | 1.97* ± 0.01 |
| **BuoH** | 1.01* ± 0.057 | 1.02* ± 0.05 | 1.30* ± 0.01 | 1.06* ± 0.01 |
| **Adenosine (7μg/mL)** | 0.95* ± 0.067 | 1.03** ± 0.29 | 1.67* ± 0.21 | 1.039* ± 0.01 |
| **Tryptophan (6μg/mL)** | 1.39* ± 0.054 | 1.53** ± 0.21 | 2.41* ± 0.08 | 2.005* ± 0.005 |
| **Piroxicam (10μg/mL)** | 0.99* ± 0.001 | 0.98* ± 0.07 | 1.90* ± 0.07 | 1.13* ± 0.14 |

**^#^ Data are expressed as the mean ± SD (n = 3). ^#^p< 0.001, *p< 0.001, **p < 0.01 indicates statistically significant difference compared to the control group and LPS group using One way ANOVA test.**
